# Six Amino Acid Residues in a 1200 *Å*2 Interface Mediate Binding of Factor VIII to an IgG4κ Inhibitory Antibody

**DOI:** 10.1371/journal.pone.0116577

**Published:** 2015-01-23

**Authors:** Jasper C. Lin, Ruth A. Ettinger, Jason T. Schuman, Ai-Hong Zhang, Muhammad Wamiq-Adhami, Phuong-Cac T. Nguyen, Shelley M. Nakaya-Fletcher, Komal Puranik, Arthur R. Thompson, Kathleen P. Pratt

**Affiliations:** 1 Puget Sound Blood Center Research Institute, Seattle, Washington, United States of America; 2 Division of Hematology, Dept. Medicine, University of Washington, Seattle, Washington, United States of America; 3 General Electric (GE) Healthcare Life Sciences, Portland, Oregon, United States of America; 4 Uniformed Services University of the Health Sciences, Bethesda, Maryland, United States of America; 5 Department of Pathology, Walter Reed National Military Medical Center, Bethesda, Maryland, United States of America; Emory University School of Medicine, UNITED STATES

## Abstract

The development of neutralizing anti-factor VIII (FVIII) antibodies complicates the treatment of many hemophilia A patients. The C-terminal C2 domain is a particularly antigenic FVIII region. A crystal structure of recombinant FVIII-C2 bound to an Fab fragment of the patient-derived monoclonal antibody BO2C11, which recognizes an immunodominant inhibitor epitope on FVIII and blocks its ability to bind von Willebrand factor (VWF) and phospholipids, revealed that 15 amino acids in FVIII contact this antibody. Forty-three recombinant FVIII-C2 proteins, each with a surface-exposed side chain mutated to alanine or another residue, were generated, and surface plasmon resonance studies were carried out to evaluate effects of these substitutions on BO2C11/FVIII-C2 binding affinity. Thermodynamic analysis of experiments carried out at three temperatures indicated that one beta hairpin turn at the antigen-antibody interface (FVIII-F2196, N2198, M2199 and F2200) plus two non-contiguous arginines (FVIII-R2215 and R2220), contributed appreciably to the affinity. B-domain-deleted (BDD) FVIII-F2196A, FVIII-F2196K and FVIII-M2199A were generated and characterized. Their pro-coagulant activities and binding to VWF were similar to those of WT-BDD-FVIII, and FVIII-F2196K avoided neutralization by BO2C11 and murine inhibitory mAb 1B5. This study suggests specific sites for amino acid substitutions to rationally design FVIII variants capable of evading immunodominant neutralizing anti-FVIII antibodies.

## Introduction

The most prevalent complication encountered when hemophilia A patients receive infusions of factor VIII (FVIII) is the development of antibodies that neutralize its pro-coagulant function. These “inhibitor” antibodies develop in ~25–30% of severe hemophilia A patients [1, 2]. They can also occur in individuals with mild or moderate hemophilia A [3] and in non-hemophilic individuals who develop immunity to their endogenous FVIII [4]. The resulting bleeding disorders can be difficult and extremely expensive to treat [5]. There is a compelling need for improved therapies to reduce the incidence of inhibitors and to provide effective alternative treatments when they do occur.

FVIII is a highly immunogenic molecule, as evidenced by the development of anti-FVIII antibodies in both humans [1, 6] and animals [7–9] following infusion with therapeutic levels of FVIII. FVIII consists of three A domains that are homologous to ceruloplasmin [10], a B domain with no close homologues identified, and two C domains that are members of the discoidin family [11], arranged as follows: A1-A2-B-A3-C1-C2 [12]. Antibodies that bind to functionally important regions, e.g. surfaces where thrombin or activated factor X bind to FVIII and activate it proteolytically, or where FVIII attaches to platelet membranes, von Willebrand factor (VWF) or components of the intrinsic factor X activating complex, constitute a subset of anti-FVIII IgGs that inhibit its cofactor activity. Identification of specific amino acids essential for formation of FVIII-IgG complexes will increase our understanding of molecular mechanisms underlying these immune reactions and indicate sites that could be modified to produce less antigenic FVIII proteins. Antibodies specific for the FVIII C2 domain (FVIII-C2) are commonly found in inhibitor patients. FVIII-C2 interacts with VWF, activated platelets, thrombin, activated factor IX and factor X [13–18].

In attempting to evaluate the feasibility of modifying specific FVIII residues to evade inhibitory antibodies, it would be helpful to know how many amino acids contribute significantly to antigen-antibody complex formation. Elegant studies by the Lollar group utilized competition ELISAs [19] plus functional analyses [19, 20] to identify five partially overlapping B-cell epitopes on FVIII-C2 [19], and subsets of these epitopes were subsequently identified by x-ray [21, 22], H/D exchange mass spectroscopy [23] and SPR-based approaches [24]. FVIII epitopes have also been mapped by analysis of hybrid or domain-deleted FVIII proteins, competitive inhibition by peptides, immunoblotting/immunoprecipitation, mass spectrometry, luminex assays and phage display [25–31]. These studies have identified functionally important sites on the FVIII-C2 surface, however there is still a need to better characterize the epitopes recognized by human antibodies, e.g. to identify the FVIII side chains that constitute “hot spots” for binding to clinically relevant human antibodies.

The monoclonal antibody (mAb) BO2C11 is an IgG_4_κ purified from an EBV-immortalized B-cell line derived from an inhibitor subject’s blood [32]. BO2C11 interferes with FVIII attachment to phospholipid membranes and VWF [32]. A crystal structure of BO2C11-Fab bound to recombinant FVIII-C2 [33] showed that the antigen-antibody interface buries ~1200 *Å* ^2^ of each molecular surface and includes hydrophobic interactions, hydrogen and ionic bonds between 15 FVIII side chains and BO2C11. Earlier studies of thermodynamic and electrostatic aspects of BO2C11 binding to WT-FVIII [34, 35] noted that this interaction involves both electrostatic and hydrophobic effects. In another recent study [24] a series of recombinant FVIII-C2 proteins was generated in which each mutant FVIII-C2 (mutein) had a single surface residue changed to alanine and/or another residue. The effects of these substitutions on binding to BO2C11-Fab were evaluated by SPR and the results confirmed [15] that the binding was mediated primarily by one of two beta-hairpin turns that contact the antibody. BO2C11-FVIII binding is characterized by a fast association with a rate constant (k_a_) whose measurement under standard conditions (25°C and 1 atm) approaches the limit of detection on a Biacore T100 instrument [24], therefore accurate calculation of changes in free energy associated with specific amino acid substitutions under these conditions requires additional data besides standard-state k_a_ and k_d_ values. The present study characterizes the affinities and thermodynamics of wild-type and mutant FVIII-C2 proteins binding to BO2C11 and identifies sites at which the amino acid sequence could be modified to avoid FVIII neutralization by this human mAb. Three B-domain-deleted (BDD) FVIII proteins were generated and characterized as proof-of-principle for the rational design of less antigenic FVIII proteins.

## Materials and Methods

### Binding kinetics

BO2C11-Fab was kindly provided by Dr. Marc Jacquemin. Anti-FVIII-A2 domain MAb413 was from the American Red Cross and anti-FVIII-C2 mAb 1B5 from Green Mountain Antibodies (Burlington, VT; clone #GMA-8008). Generation of recombinant FVIII-C2 and BDD-FVIII proteins is described in S1 Methods and S1 Table. SPR measurements were carried out on Biacore T-100 and T-200 instruments (GE Healthcare Life Sciences, Piscataway, NJ). BO2C11-Fab (3.9 mg/mL) was diluted 200-fold in 10 mM sodium acetate pH 5.0 and immobilized by amine coupling, using 1-ethyl-3*-(*3-dimethyl-aminopropyl)carbodiimide-HCl and N-hydroxysuccinimide (GE) to activate the CM5 surface, then injecting the protein serially in Running Buffer HBS-EP+ (GE) until 300±5 resonance units (RUs) were recorded, at which point 35 μl ethanolamine was injected to quench reactive sites on the chip. This immobilization level yielded Rmax values between 30 and 100RU. These relatively low values circumvented potential problems in measuring accurate kinetic constants due to mass transport limitation. A reference flow cell was created by activating and then immediately deactivating the sensor surface without exposing it to BO2C11-Fab. FVIII-C2 binding to the reference cell was not detected. FVIII-C2 muteins at 0.4 to 50 nM were injected for at least 120 sec, and dissociation was monitored for 300–900 sec to determine association and dissociation rate constants at 25°C. Regeneration was accomplished by injecting 10 mM glycine-HCl, pH 2.0 (GE) for 30–60 sec and resonance signals were monitored to ensure complete dissociation of FVIII-C2 before initiating the next experiment. The sensorgrams were subtracted from the reference cell signal, subsequently subtracted from a blank run signal and subjected to curve fit analysis using Biacore Evaluation Software version 2.0.1 with a 1:1 binding model.

### Thermodynamic analysis

Van’t Hoff analysis was carried out using the Biacore T100 Evaluation Software, version 2.0.1. On and off rates for FVIII-C2/BO2C11-Fab binding were obtained by fitting sensorgrams to a theoretical 1:1 binding curve, and the resulting kinetic constants were converted to dissociation equilibrium constants, *K_D_*=*k_d_*/*k_a_* [36]. SPR measurements were executed from 10 to 40°C in increments of 5°C for FVIII-C2 muteins that had dissociation constants greater than 4X that of WT-FVIII-C2 at 25°C. Van’t Hoff plots were generated by plotting ln *K_D_* versus 1/*T*, and thermodynamic values were obtained utilizing the relationship: ln *K_D_* = (*ΔH_A_º/RT*)−(*ΔS_A_º/R*), where *ΔH_A_º* and *ΔS_A_º* are enthalpy and entropy of the binding reaction, respectively, at standard conditions (25°C and 1 atm), *R* is the gas constant and *T* is the temperature in Kelvin. Linear data fitting allowed calculation of standard enthalpies from slopes (*ΔH_A_º/R*) and standard entropies were obtained from y-intercepts (-*ΔS_A_º/R*) [37]. Gibbs free energies (*ΔG_A_º = ΔH_A_º−TΔS_A_º*) were calculated and WT-FVIII-C2 *ΔG_A_º* was subtracted from the *ΔG_A_º* for each mutein (*ΔΔG_A_º* = *ΔG_A_º* (mutein)−*ΔG_A_º* (WT-FVIII-C2)); the resulting *ΔΔG_A_º* values estimate the energetic cost of the corresponding amino acid substitutions. Substitutions that resulted in increased free energy or enthalpy relative to the values of these parameters for WT-FVIII-C2 (*i.e. ΔΔG_A_º* or *ΔΔH_A_º* > 0) reflected a loss of binding energy or enthalpy, respectively, when the side chain was altered, while substitutions that decreased the entropy (*Δ*(*TΔS_A_º*) < 0) indicated that binding of the mutein to the BO2C11 Fab was less entropically driven than WT-FVIII-C2 binding. The data were also fit to a modified Gibbs-Helmholtz equation: ΔG(T) = ΔH_*A*_ *º*−TΔS_*A*_ *º* + ΔCp[T−T_*A*_ *º*−T_*l*_n (T/T_*A*_ *º*)], to determine if there were significant contributions to the ΔG(T) from heat capacity changes (ΔCp).

### Binding of FVIII-C2 proteins to phospholipids

WT-FVIII-C2 and the muteins with amino acid substitutions that had pronounced effects on binding to BO2C11 were tested for binding to phosphatidylserine (PS) and phosphatidylcholine (PC) membranes (PC and PS:PC = 20:80) by ELISAs (S1 Methods).

### Characterization of rationally designed BDD-FVIII proteins

WT-BDD-FVIII and BDD-FVIII muteins F2196A, F2196K and M2199A were generated using a BHK-M expression system [25, 38] kindly provided by Dr. Pete Lollar. Expression, purification and characterization are described in S1 Methods. Briefly, >20 lines/protein were expanded during stable selection and evaluated for BDD-FVIII expression by ELISA. BDD-FVIII proteins were purified from the highest expressing cell lines by ion-exchange chromatography. A Biacore T200 instrument (GE) was used to monitor the binding kinetics between purified BDD-FVIII proteins and immobilized BO2C11 as well as VWF, using a modification of recently published protocols [39, 40]. The ability of neutralizing anti-FVIII-C2 mAbs BO2C11 and 1B5, and of neutralizing anti-FVIII-A2 MAb413, to inhibit the activity of wild-type and mutant BDD-FVIII proteins was evaluated using modified Bethesda [41] assays, with FVIII activity measured by both chromogenic and one-stage coagulation assays.

## Results

### Binding kinetics

**Figure 1:**
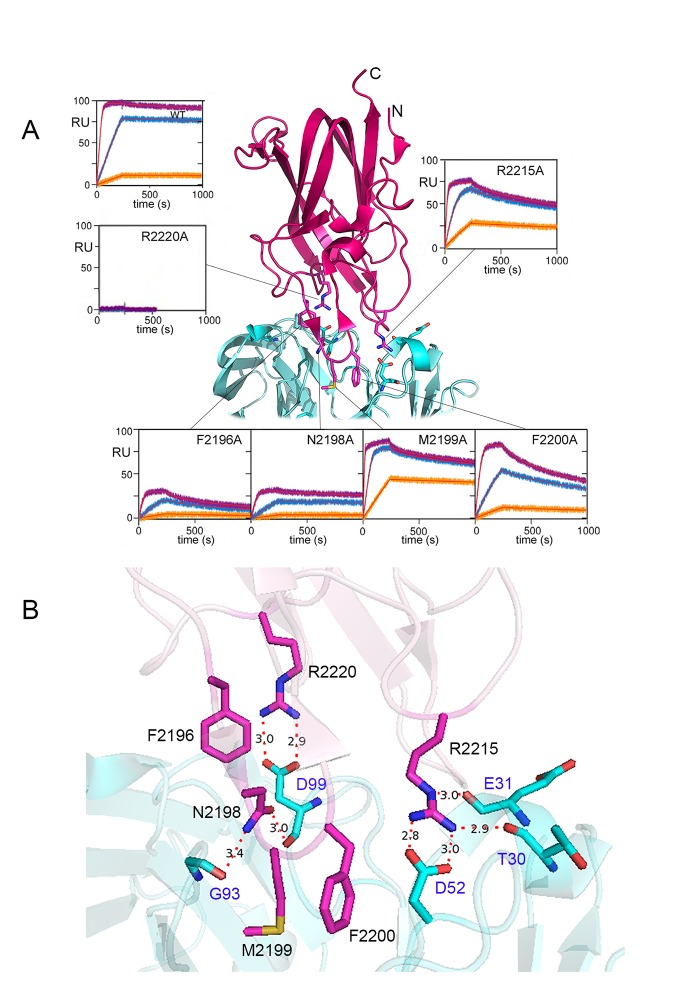
Sensorgrams of FVIII-C2 mutants that bound BO2C11 with reduced affinity. **A.** The crystal structure of FVIII-C2 (hot pink) complexed with the BO2C11 Fab (cyan) [33] is shown in cartoon representation and with relevant side chains shown explicitly. Sensorgrams corresponding to muteins that bound BO2C11 with k_d_ > 4.0X that of WT-FVIII-C2 are shown; black lines map each sensorgram to the relevant WT-FVIII-C2 residue. The sensorgrams record the mass of the FVIII-C2 protein that becomes attached to the Fab-coated chip. The orange, blue and purple curves show sensorgrams generated following injections of increasing concentrations of the indicated FVIII-C2 protein. The signals are measured in Resonance Units (RUs), which are arbitrary units. Fits to theoretical curves generated using the experimentally derived rate constants are shown in red. Larger images of these sensorgrams are in S1 Fig. Note that residues 2198–2201 comprise a beta turn that adopted slightly different conformations in the crystal structures of FVIII-C2 [50] and of FVIII-C2 bound to BO2C11 [33]. ***B.*** Expanded view of the FVIII-C2 (hot pink)—BO2C11 (cyan) interface. The six FVIII-C2 residues comprising the functional epitope are shown in stick representation (labeled in black font), as are the BO2C11 residues that form hydrogen bonds with the side chains of these six residues (labeled in blue font). The corresponding proteins are shown in light, transparent cartoon representation. Hydrogen bonds are indicated by red dotted lines and the interatomic distances are given.

Five SPR runs were carried out to determine average kinetic constants for WT-FVIII-C2 binding to BO2C11-Fab. The kinetic constants were the same (within a factor of 2) for association measurement periods of 120 or 300 sec and for dissociation measurement periods of 900 or 1800 sec. Average *k_a_* and *k*_d_ for WT-FVIII-C2 were 9.4x10^6^ *M^-1^s^-1^* and 8.5x10^-5^ *s^-1^*, respectively. Kinetic constants for the mutants are found in Table 1. R2220A and R2220Q muteins showed virtually no binding to BO2C11-Fab (Fig. 1). Curve fits for these mutants revealed a maximum resonance signal (Rmax)≤12% of the Rmax for WT-FVIII-C2. All other muteins had Rmax levels similar to that of WT-FVIII-C2, indicating they were capable of binding BO2C11. However, some dissociation rate constants were considerably higher than the *k_d_* for WT-FVIII-C2 (Table 1). An initial screen of the data using a cutoff criterion of *k*_d_(WT)/*k*_d_(mutein) > 2.0 identified nine residues as potential B-cell epitope residues, in addition to R2220. Visual inspection of the crystal structures revealed that with the exception of Q2316A, these alanine substitution sites were distant from the cluster of residues whose substitution had a more significant impact on the kinetics. Therefore, to minimize the inclusion of outlier residues, a more stringent criterion of *k*_d_(WT)/*k*_d_(mutein) > 4.0 was applied, leading to the identification of five residues meeting this criterion plus R2220, whose substitution to alanine or glutamine completely abrogated binding to BO2C11.

**Table 1:** Kinetic and equilibrium constants for binding of WT and mutant FVIII-C2 proteins to BO2C11.

	**FVIII-C2 variant**	k_a_(SE) * *(M^-1^ s^-1^*)	***k_d_*(SE)(*s^-1^*)**	***K_D_* = k_d_/k_a_ (pM)**	**k_d_(mutein)/ k_d_(WT)**	**residence time (hr)**	**dissociation half-life (min)**
1	WT-FVIII-C2	9.4 × 10^6^ (2.7 × 10^5^)	8.5 × 10^-5^ (2.5 × 10^-6^)	9	1.0	3.3	136
2	S2173I	1.5 × 10^7^ (2.7 × 10^5^)	1.1 × 10^-4^ (2.9 × 10^-6^)	7	1.3	2.5	105
3	E2181A	2.2 × 10^7^ (3.8 × 10^5^)	1.9 × 10^-4^ (2.9 × 10^-6^)	9	2.2	1.5	61
**4**	**F2196A^†‡^**	**1.7 × 10^7^** (**2.3 × 10^5^**)	**2.5 × 10^-3^** (**2.0 × 10^-5^**)	**147**	29.4	0.1	5
**5**	**T2197A**	**4.4 × 10^6^** (**.6 × 10^4^**)	**4.3 × 10^-5^** (**4.4 × 10^-7^**)	**10**	0.5	6.5	269
**6**	**N2198A^†^**	**6.2 × 10^6^** (**3.4 × 10^4^**)	**3.5 × 10^-4^** (**2.3 × 10^-4^**)	**56**	4.1	0.8	33
**7**	**M2199A^†^**	>3.0 × 10^7^	**7.6 × 10^-4^** (**6.9 × 10^-6^**)		8.9	0.4	15
**8**	**F2200A^†‡^**	**9.4 × 10^6^** (**3.6 × 10^4^**)	**2.3 × 10^-3^** (**7.9 × 10^-6^**)	**240**	27.1	0.1	5
9	A2201P	8.4 × 10^6^ (5.8 × 10^4^)	1.6 × 10^-4^ (1.6 × 10^-6^)	19	1.9	1.7	72
10	T2202A	6.0 × 10^6^ (2.9 × 10^4^)	3.5 × 10^-5^ (1.5 × 10^-7^)	6	0.4	7.9	330
11	K2207A	4.6 × 10^6^ (7.0 × 10^3^)	5.9 × 10^-5^ (1.7 × 10^-7^)	13	0.7	4.7	196
12	H2211A	7.1 × 10^6^ (2.3 × 10^4^)	3.8 × 10^-5^ (2.9 × 10^-7^)	5	0.4	7.3	304
13	L2212A	2.3 × 10^7^ (2.3 × 10^5^)	4.4 × 10^-5^ (2.6 × 10^-7^)	2	0.5	6.3	263
14	Q2213A	1.4 × 10^7^ (2.6 × 10^4^)	6.1 × 10^-5^ (3.1 × 10^-7^)	4	0.7	4.6	189
**15**	**R2215A^†^**	**4.1 × 10^6^** (**1.2 × 10^4^**)	**6.1 × 10^-4^** (**1.3 × 10^-6^**)	**150**	7.2	0.5	19
**16**	**R2220A^†‡^**	**NB**	**NB**	**NB**	NB	NB	NB
**17**	**R2220Q^†‡^**	**NB**	**NB**	**NB**	NB	NB	NB
**18**	**Q2222A**	**7.4 × 10^6^** (**1.7 × 10^5^**)	**3.9 × 10^-5^** (**2.0 × 10^-7^**)	**5**	0.5	7.1	296
**19**	**V2223M**	**5.7 × 10^6^** (**1.6 × 10^5^**)	**3.6 × 10^-5^** (**1.6 × 10^-7^**)	**6**	0.4	7.7	321
20	N2224A	>3.0 × 10^7^	8.6 × 10^-5^ (5.8 × 10^-7^)		1.0	3.2	134
21	N2225A	1.6 × 10^7^ (1.3 × 10^5^)	3.9 × 10^-5^ (2.2 × 10^-7^)	2	0.5	7.1	296
22	K2227A	1.9 × 10^7^ (8.6 × 10^4^)	6.4 × 10^-5^ (4.0 × 10^-7^)	3	0.8	4.3	181
23	K2227Q	6.8 × 10^6^ (2.9 × 10^4^)	2.0 × 10^-5^ (3.1 × 10^-7^)	3	0.2	13.9	578
24	K2249A	6.1 × 10^6^ (1.8 × 10^4^)	3.7 × 10^-5^ (2.6 × 10^-7^)	6	0.4	7.5	312
**25**	**S2250A**	**4.3 × 10^6^** (**1.9 × 10^4^**)	**9.4 × 10^-5^** (**3.1 × 10^-7^**)	**22**	1.1	3.0	123
**26**	**L2251A**	**8.1 × 10^6^** (**2.3 × 10^5^**)	**5.0 × 10^-5^** (**2.4 × 10^-7^**)	**6**	0.6	5.6	231
**27**	**L2252A**	**2.8 × 10^7^** (**1.1 × 10^5^**)	**1.7 × 10^-4^** (**6.3 × 10^-7^**)	**6**	2.0	1.6	68
**28**	**T2253A**	**5.3 × 10^6^** (**3.4 × 10^4^**)	**5.2 × 10^-5^** (**1.4 × 10^-7^**)	**10**	0.6	5.3	222
29	H2269A	2.2 × 10^7^ (4.7 × 10^5^)	1.7 × 10^-4^ (3.4 × 10^-6^)	8	2.0	1.6	68
30	Q2270A	>3.0 × 10^7^	1.8 × 10^-4^ (1.7 × 10^-5^)		2.1	1.5	64
31	T2272A	1.3 × 10^7^ (4.6 × 10^4^)	5.4 × 10^-5^ (2.3 × 10^-7^)	4	0.6	5.1	214
32	L2273A	1.1 × 10^7^ (1.4 × 10^5^)	1.7 × 10^-4^ (2.6 × 10^-6^)	15	2.0	1.6	68
33	N2277A	1.2 × 10^7^ (3.0 × 10^4^)	6.0 × 10^-5^ (4.8 × 10^-7^)	5	0.7	4.6	193
34	K2279A	7.2 × 10^6^ (8.2 × 10^4^)	1.9 × 10^-4^ (2.8 × 10^-6^)	26	2.2	1.5	61
35	P2300L	1.8 × 10^7^ (1.9 × 10^5^)	9.5 × 10^-5^ (7.1 × 10^-7^)	5	1.1	2.9	122
36	L2302A	2.4 × 10^7^ (1.9 × 10^5^)	5.7 × 10^-5^ (4.7 × 10^-7^)	2	0.7	4.9	203
37	R2304H	1.0 × 10^7^ (2.4 × 10^5^)	9.2 × 10^-5^ (4.5 × 10^-6^)	9	1.1	3.0	126
38	R2307Q	1.9 × 10^7^ (1.4 × 10^5^)	9.0 × 10^-5^ (5.2 × 10^-7^)	5	1.1	3.1	128
39	H2309A	1.5 × 10^7^ (7.0 × 10^4^)	2.9 × 10^-5^ (1.4 × 10^-7^)	2	0.3	9.6	398
40	Q2311A	2.3 × 10^7^ (1.4 × 10^5^)	6.8 × 10^-5^ (3.6 × 10^-7^)	3	0.8	4.1	170
41	W2313Y	8.2 × 10^6^ (1.2 × 10^5^)	8.7 × 10^-5^ (2.8 × 10^-6^)	11	1.0	3.2	133
**42**	**H2315A**	**1.5 × 10^7^** (**8.8 × 10^4^**)	**7.3 × 10^-5^** (**2.7 × 10^-7^**)	**5**	0.9	3.8	158
**43**	**Q2316A**	**1.3 × 10^7^** (**3.5 × 10^5^**)	**3.2 × 10^-4^** (**6.1 × 10^-7^**)	**25**	3.8	0.9	36
44	E2327A	2.1 × 10^7^ (1.4 × 10^5^)	5.4 × 10^-5^ (3.3 × 10^-7^)	3	0.6	5.1	214

Based on the good agreement between the theoretical Rmax (determined using the GE Biacore T100 software) and the measured Rmax values, a 1:1 binding model was determined to be appropriate for these experiments. Occasionally, the sensorgram corresponding to the highest concentration was omitted from analysis due to apparent non-specific binding, but in these cases the binding curves at lower FVIII-C2 concentrations were adequate to calculate reliable rate constants. The entries in bold are the 16 substitutions at 15 positions at the BO2C11 binding interface (“contacts” defined as FVIII-C2-BO2C11 interatomic d < 3.8 Å). Residence time = 1/k_d_. Dissociation half-life = ln 2 /k_d_. *SE = Standard Error of the curve fit calculated by the Biacore analysis software. ^†^These substitutions resulted in a k_d_ >4X that of WT-FVIII-C2 for BO2C11 or abrogated binding to BO2C11. ^‡^These substitutions decreased the residence time for the FVIII-C2-mutein/BO2C11 complex at least 10X. The kinetic range for the Biacore T100 is: k_a_ constants from ~10^3^–10^7^ M^-1^s^-1^; k_d_ constants from ~10^-5^–0.5 s^-1^. For fast associations with fitted k_a_ ≥ 3.2E+06, the evaluation software warns that the value approaches the limit that can be measured by the instrument. All k_a_ values between 1.0E+07 and 3.0E+07 and kinetic data calculated using these k_a_ values are underlined. All fitted k_a_ values >3.0E+07 are indicated as “>3.0E+07” and the corresponding K_D_ values were not calculated. For slow dissociations with fitted k_d_ ≤ 3.0E-05, the evaluation software warns that the value approaches the limit that can be measured by the instrument. All k_d_ values between 1.0E-05 and 3.0E-05 and kinetic data calculated using these k_d_ values are underlined.

In order to test whether muteins with altered binding kinetics to BO2C11 were misfolded, their binding to additional inhibitory anti-FVIII-C2 mAbs was evaluated by SPR. All bound to 1–8 inhibitory mAbs with affinities similar to that of WT-FVIII-C2 [24], indicating that these substitutions did not cause global misfolding. Representative sensorgrams depicting the binding of one of these mAbs (anti-FVIII-C2 mAb I54) to the 6 FVIII-C2 muteins that comprise the functional epitope for BO2C11 is shown in S1 Fig.

### Thermodynamic analysis

**Figure 2:**
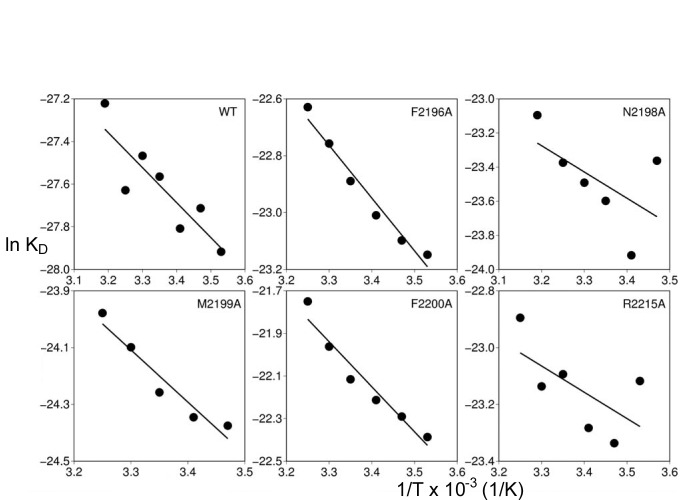
Van’t Hoff plots of FVIII-C2 mutants that dissociated faster from BO2C11. Van’t Hoff plots were generated from SPR runs carried out at several temperatures for WT-FVIII-C2 and mutants FVIII-C2-F2196A, FVIII-C2-N2198A, FVIII-C2-M2199A, FVIII-C2-F2200A and FVIII-C2-R2215A. For many of the experiments, the value for *k*_a_ at 40°C or for *k*_d_ at 10°C approached or exceeded the sensitivity limit for accurately fitting the sensorgrams. These outlier data points were not included in the corresponding thermodynamic analyses. See Table 2 for additional data including the thermodynamic values obtained from linear regression analysis.

The five FVIII-C2 muteins that met the cutoff criterion and had measurable k_d_ values had calculated antigen-antibody binding half-life values from 4–33 minutes, compared to 136 minutes for WT-FVIII-C2 (Table 1). SPR measurements carried out from 10–40°C in 5°C increments confirmed that substitutions F2196A, N2198A, M2199A, F2200A and R2215A destabilized the BO2C11-Fab/FVIII-C2 complex (Table 2 and Fig. 2), with F2200A having the most pronounced effect on the binding free energy (*ΔΔG_A_º* = 13 kJ/mol). Thermodynamic experiments carried out for four additional muteins with alanine substitutions at the antigen-antibody interface (T2197A, S2250A, L2251A and L2252A) indicated these residues each contributed <10 kJ/mol to the affinity. The sum of individual *ΔΔG_A_º* values for the alanine substitutions F2196A, N2198A, M2199A, F2200A and R2215A was calculated and compared to the measured *ΔG_A_º* (-68±1 kJ/mol) for WT-FVIII-C2 binding to see how well the summed contributions accounted for the overall binding energy. The sum indicated that these residues contributed approximately 53 kJ/mol, consistent with the importance of the salt link between FVIII-R2220 and BO2C11-D120, whose energetic contribution could not be measured directly. Nonlinear fitting of the WT and mutant data to a three-parameter equation (with *Δ*H, *Δ*S and *Δ*Cp as fitting parameters) indicated that the *Δ*Cp (changes in heat capacity) values were all under 4kJ/mol and thus did not contribute significantly to the *Δ*G values (S2 Table). Therefore, the linear van’t Hoff analysis described in Materials and Methods was considered adequate to fit these data.

**Table 2:** Thermodynamic values from van’t Hoff analysis.

**FVIII-C2 variant**	***ΔH_A_º^†^***	***TΔS_A_º^†^***	***ΔG_A_º^‡,§^***	***ΔΔH_A_º***	***Δ*(*TΔS_A_º*)**	***ΔΔG_A_º^§^***	***K_D_º*(pM)**	***K_D_* (pM) =*k_d_*/*k_a_^||^***
WT-FVIII-C2	-14 ± 3	54 ± 3	-68 ± 1	N.A.	N.A.	N.A.	1	9
F2196A*	**-18 ± 7**	**38 ± 7**	**-56 ± 1**	**-4 ± 8**	**-16 ± 8**	**12 ± 1**	**154**	**147**
T2197A	-12 ± 5	52 ± 5	-64 ± 1	2 ± 8	-2 ± 8	4 ± 1	6	10
**N2198A**	**-13 ± 9**	**45 ± 9**	**-58 ± 1**	**1 ± 10**	**-9 ± 10**	**10 ± 1**	**68**	**56**
**M2199A**	**-16 ± 2**	**44 ± 2**	**-60 ± 1**	**-2 ± 4**	**-10 ± 4**	**8 ± 1**	**30**	
**F2200A**	**-18 ± 2**	**37 ± 2**	**-55 ± 1**	**-4 ± 4**	**-17 ± 4**	**13 ± 1**	**230**	**240**
**R2215A**	**-8 ± 5**	**49 ± 5**	**-57 ± 1**	**6 ± 5**	**-5 ± 5**	**11 ± 1**	**100**	**150**
S2250A	-3 ± 5	57 ± 5	-60 ± 1	11 ± 5	3 ± 5	8 ± 1	30	22
L2251A	-31 ± 10	35 ± 10	-66 ± 1	-17 ± 11	-19 ± 11	2 ± 1	3	6
L2252A	-49 ± 12	14 ± 12	-63 ± 1	-35 ± 12	-40 ± 12	5 ± 1	9	6

Data are in kJ/mol unless otherwise specified.

*Bold-face regions indicate substitutions at the *functional* epitope for BO2C11, i.e. where the substitution caused a greater than fourfold increase in *k_d_* relative to WT-FVIII-C2 binding.

^†^The standard errors (SE) of ΔH_***A***_º and TΔS_***A***_ºare based on the SE of the slope and intercept, respectively.

Reported errors for ΔΔH_A_º, Δ(TΔS_A_º) and ΔΔG_A_º^§^ are the square roots of the sums of the squares of the errors.

^‡^The SE of ΔG***Aº*** was derived from the SE of fitted values from the linear regression (van’t Hoff analysis), transformed to the ΔG***Aº*** scale (e.g. SE (ln(*K_D_*)/T = 298)xRT/1000)

^§^The ΔG_***A***_º and ΔΔG_***A***_º errors were all less than one kJ/mol but are reported here as “±1” for consistency with the significant figures of the measured data.

^||^The *K_D_* values from the earlier kinetic runs at 25^o^C only (final column) are included for comparison with *K_D_* values derived from the *ΔG_A_º*of SPR runs carried out at several temperatures.

### Binding of FVIII-C2 proteins to phospholipids

WT-FVIII-C2 and C2 muteins F2196A and M2199A bound to PS-containing membranes in a dose-dependent manner (S2 Fig.), consistent with results of similar solid-phase assays reported earlier for FVIII-C2 [42], whereas muteins N2198A, F2200A, R2215A and R2220A did not.

### Characterization of rationally designed BDD-FVIII proteins

The two substitutions that destabilized the Fab-FVIII-C2 interaction without abrogating binding to negatively charged phospholipids, F2196A and M2199A, were introduced into BDD-FVIII to assess their effect on FVIII function, and FVIII-F2196K was also produced. Cell lines with expression levels similar to that of WT-BDD-FVIII were isolated for BDD-FVIII-F2196K and BDD-FVIII-M2199A. The highest expressing F2196A cell lines showed ~10-fold lower expression levels than the other lines at similar confluencies (S3 Fig.). All FVIII mutein cell lines showed specific activities in cell supernatants similar to WT-BDD-FVIII in one-stage coagulation and chromogenic assays (Table 3 and S3 Fig.). Activation quotients (ratios of FVIII activities in a two-stage coagulation assay divided by one-stage assay activities, indicating the amount of pre-activated FVIII and thus affecting subsequent activity measurements) were nearly identical to that of WT-BDD-FVIII.

**Table 3:** Mean specific activity measurements and activation quotients for BDD-FVIII muteins in cell supernatants.

A. One-stage coagulation assay				
	Specific Activity (IU/mg)*	SD^†^	% WT	% WT SD
WT-BDD-FVIII	3300	990	100	42
F2196A-BDD-FVIII	2600	200	79	24
F2196K-BDD-FVIII	4300	1700	130	65
M2199A-BDD-FVIII	4000	720	120	42
B. Activation quotient				
	Activation Quotient* ^,‡^	SD^†^	% WT	% WT SD
WT-BDD-FVIII	25	3	100	16
F2196A-BDD-FVIII	25	6	100	25
F2196K-BDD-FVIII	26	7	100	29
M2199A-BDD-FVIII	26	4	100	19
C. Chromogenic assay				
	Specific Activity (IU/mg) *	SD^†^	% WT	% WT SD
WT-BDD-FVIII	2700	1200	100	63
F2196A-BDD-FVIII	1900	300	70	33
F2196K-BDD-FVIII	2200	610	81	43
M2199A-BDD-FVIII	2700	120	100	45

IU = International FVIII Units. Activity was normalized to the FVIII expression level measured by ELISA.

*Mean values for activity and activation quotient. Activity assays were carried out using 2–3 of the highest-expressing lines chosen from 22–45 clones per mutein that were evaluated for FVIII expression levels.

^†^Standard deviation (SD) of the mean specific activity or activation quotient.

^‡^Ratio of two-stage coagulation activity divided by one-stage coagulation activity.

**Figure 3:**
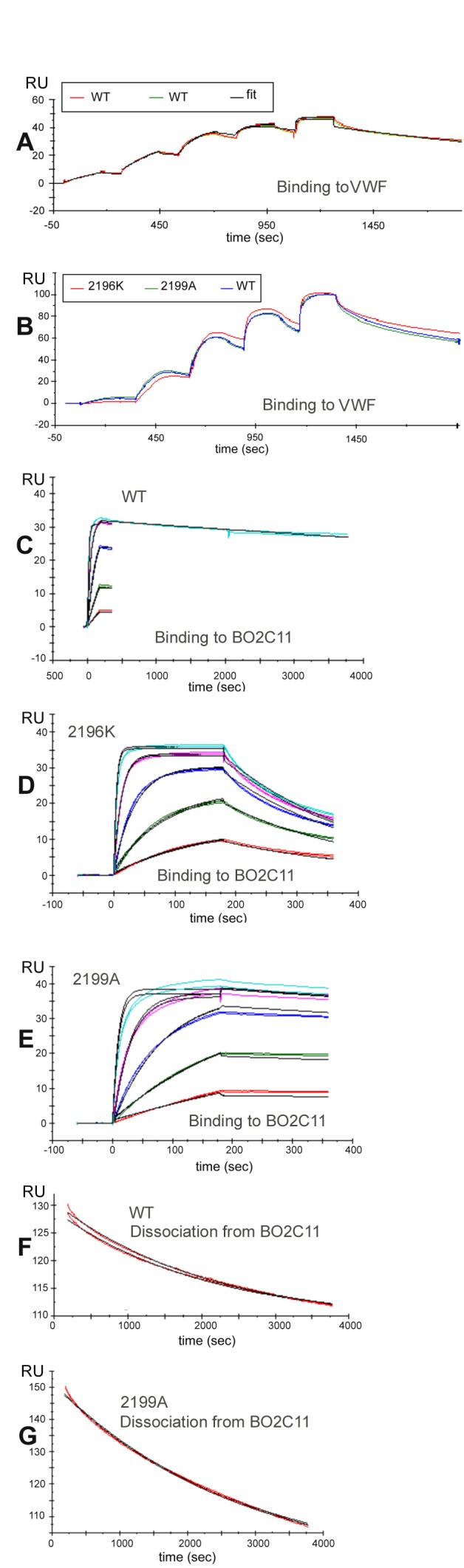
Binding of BDD-FVIII muteins to VWF (A-B) and to BO2C11 (C-G). A. VWF was attached to a CM4 chip by amine coupling at low density (200 RUs). BDD-FVIII proteins were injected in a 3-fold concentration series from 0.22–18 nM using single-cycle kinetics. Sensorgrams of duplicate SPR runs of WT-BDD-FVIII (red & green) and the fit (black) to a 1:1 model for the binding of BDD-FVIII to VWF. **B.** Representative SPR run showing overlaid normalized binding curves for WT-BDD-FVIII and variants F2196K and M2199A to VWF. **C.** BO2C11-Fab was attached to a CM5 chip by amine coupling to 20 RUs. WT-BDD-FVIII was injected at four concentrations to calculate the k_a_ for BO2C11-Fab, and the final injection was monitored for 4000s to obtain an accurate k_d_. The final experimental curve was then fitted to a theoretical curve generated using kinetic constants obtained from the separate SPR experiment shown in panel F. **D.** Five concentrations of BDD-FVIII-F2196K were injected over the BO2C11-Fab biosensor surface. This mutein showed a ~100X faster off-rate and a 28-fold decreased affinity for BO2C11-Fab compared to WT-BDD-FVIII. **E.** Five concentrations of BDD-FVIII-M2199A were injected over the BO2C11-Fab biosensor surface with the highest concentration monitored for 3600s to obtain an accurate k_d_. (The figure shows only the first ~3 minutes of the dissociation). The experimental curves were then fit to theoretical curves generated using kinetic constants obtained from the separate SPR experiment shown in panel G. **F.** BO2C11-Fab was attached to a CM5 chip by amine coupling to 70 RUs in order to obtain independent measurements of slow dissociations. The ligand (BO2C11) was included in the running buffer at 1 nM (~100x the K_D_) to prevent re-binding and a high flow rate of 100 µl/min was used to avoid mass transport limitations. The dissociation of WT-BDD-FVIII from BO2C11-Fab was measured for >1 hr. Red = experimental curves, black = fit to curve calculated using a 1:1 binding model. The injection and association phases are not pictured. **G.** The dissociation of BDD-FVIII-M2199A from the lower-density BO2C11-Fab surface was measured for >1 hr to obtain an accurate k_d_ constant for this slow dissociation. Red = experimental curves, black = fit to curve calculated using a 1:1 binding model. The injection and association phases are not pictured. (Note: the dissociation of BDD-FVIII-F2196K was considerable faster (see panel E), so a longer monitoring period was not required to obtain an accurate k_d_ for this antigen-antibody pair).

WT-BDD-FVIII and the F2196K and M2199A muteins were purified to a level similar to research-grade Kogenate-FS (specific activity: 3600 IU/mg) as assessed by gel electrophoresis and staining (S4 Fig.). These purified proteins had high specific activities of 12,000 IU/mg (WT), 9,500 IU/mg (F2196K) and 9,400 IU/mg (M2199A) by one-stage coagulation assay and 8,500 IU/mg (WT), 9,600 IU/mg (F2196K), and 13,000 IU/mg (M2199A) by chromogenic assay. SPR experiments measuring the binding kinetics of purified BDD-FVIII-F2196K and BDD-FVIII-M2199A to VWF indicated that their affinities were similar to that of WT-BDD-FVIII (K_D_ 200–290 pM), whereas these substitutions decreased the binding affinity for BO2C11 by 28X and 4X, respectively, and increased the off-rate constant for BO2C11 by 94X and 7X, respectively (Table 4 and Fig. 3).

**Table 4:** Kinetic and equilibrium constants for binding of WT-BDD-FVIII and BDD-FVIII mutein proteins to VWF and BO2C11.

**Complex**			*k*_a_ (SE) *(M^-1^ s^-1^)	***k_d_* (SE) (*s^-1^*)**	***K_D_*=*k_d_*/*k_a_ (pM*)**
1	WT-BDD-FVIII/VWF		2.4 x10^6^ (1.8 × 10^4^)	6.9 x10^-4^ (1.8 × 10^-4^)	290
2	BDD-FVIII-F2196K/VWF		2.9 x10^6^ (3.3 × 10^4^)	5.4 x10^-4^ (6.7 × 10^-6^)	200
3	BDD-FVIII-M2199A/VWF		2.7 x10^6^ (1.8 × 10^4^)	6.7 x10^-4^ (7.5 × 10^-6^)	250
4	WT-BDD-FVIII/BO2C11		2.3 x10^6^ (1.2 × 10^3^)	4.8 x10^-5^ (5.6 × 10^-8^)	21
5	BDD-FVIII-F2196K/BO2C11		7.8 x10^6^ (1.9 × 10^4^)	4.5 x10^-3^ (9.5 × 10^-6^)	580
6	BDD-FVIII-M2199A/BO2C11		3.5 x10^6^ (1.8 × 10^3^)	3.2 x10^-4^ (2 × 10^-7^)	92

*Kinetic and equilibrium constants were calculated assuming a 1:1 stoichiometry for binding of FVIII-C2 proteins to the immobilized BO2C11 Fab fragment or to VWF.

**Figure 4:**
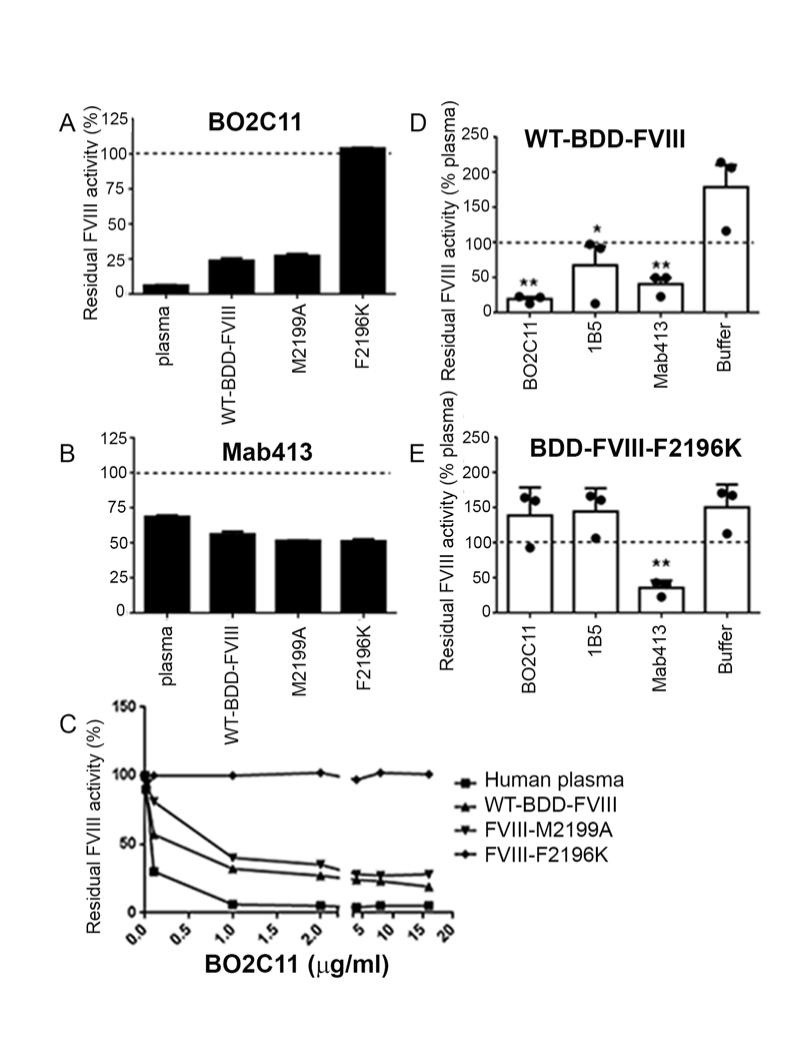
Effects of specific amino acid substitutions on antibody neutralization of BDD-FVIII muteins. *Chromogenic assays (A-C):* The BDD-FVIII proteins (~1U/ml) were mixed with either BO2C11 (*A* and *C*) or MAb413 (*B*) at a 1:1 (vol:vol) ratio and incubated at 37°C for 2 hrs. Pooled normal human citrated plasma was included as an additional control. The FVIII activity was then measured using the chromogenic Coatest SP4 FVIII functional assay. The FVIII activities for WT-BDD-FVIII and for the M2199A and F2196K variants in the absence of inhibitory mAbs were adjusted to 100% (dotted lines in *A* and *B*) and the residual FVIII activities shown in A-C indicate the % activities of the proteins compared to their respective activities in the absence of antibodies. **A.** The residual FVIII activity in the presence of 2 µg/ml BO2C11. **B.** The residual FVIII activity in the presence of 2 µg/ml MAb413. **C.** Dose-response inhibition assays in which 0.01 µg/ml—16 µg/ml BO2C11 were added to pooled normal plasma and to the indicated BDD-FVIII proteins (1 U/ml each). *One-stage coagulation assays (D-E):* Equal volumes (300 µl) of BDD-FVIII proteins (diluted to a calculated concentration of 2 U/ml in human type AB serum; note this protein concentration was 2X that used in **A-C** in order to better visualize differences in FVIIl activity in the presence of saturating inhibitory antibodies) and 2 µg/ml of neutralizing anti-FVIII mAbs were mixed and incubated at 37°C for 2 hrs before measuring the FVIII activity. Control samples were pre-incubated with buffer instead of antibody. Residual FVIII activities were normalized to the 1-stage clotting activity measured for normal pooled human plasma (1 U/ml, dotted line). Because the assays were carried out using 2X the usual FVIII protein concentrations, the values obtained for the proteins in buffer were ~2X that for normal pooled plasma, as expected. ***D.*** One-stage coagulation assays for WT-BDD-FVIII (2 U/ml) pre-incubated with saturating amounts of antibodies BO2C11, 1B5 or Mab413, or pre-incubated with buffer alone. ***E.*** One-stage coagulation assays for BDD-FVIII-2196K (2 U/ml) pre-incubated with saturating amounts of antibodies BO2C11, 1B5 and Mab413, or pre-incubated with buffer alone. In contrast to BDD-WT-FVIII, BDD-FVIII-F2196K was not significantly inhibited by either BO2C11 or 1B5, both of which recognize an epitope on the FVIII-C2 domain that includes F2196 [24]. The inhibitory effect of the anti-FVIII-A2 domain mAb MAb413 on BDD-FVIII-F2196K was not affected by the C2 domain mutation F2196K, as expected. Data are expressed as mean ± SE. Results were analyzed using a one-tailed Student’s *t* test. *p < 0.05 and **p < 0.01 as compared to the buffer control.

Purified BDD-FVIII muteins F2196K and M2199A were tested using chromogenic and one-stage clotting assays to determine if B-cell epitope modification affected their inhibition by neutralizing monoclonal antibodies BO2C11, 1B5 or Mab413 (Fig. 4). BDD-FVIII-F2196K retained its activity in the presence of up to 16 µg/ml of BO2C11 (Fig. 4C), equivalent to ~272 Bethesda units/ml, and in the presence of saturating mAb 1B5 (Fig. 4E), which recognizes an epitope that includes F2196 [24]. A slight but consistent protection against BO2C11 inhibition was observed for BDD-FVIII-M2199A. Control experiments showed that all BDD-FVIII samples tested were inhibited to a similar extent by MAb413, a murine mAb specific to the FVIII A2 domain (Fig. 4).

## Discussion

The present study evaluates an antigenic site on FVIII recognized by a human-derived inhibitory monoclonal IgG, BO2C11. A crystal structure of FVIII-C2 bound to the BO2C11-Fab provides a detailed characterization of this human inhibitor epitope [33]. Although this structure shows which FVIII residues interact with the antibody, the contributions of particular residues to the overall interaction must be determined experimentally. In this study, each of the 15 FVIII-C2 side chains at the FVIII-C2-Fab interface was substituted to alanine or another amino acid. Antigen-antibody residence times and calculated half-lives for the complexes were derived from the measured k_d_ values under standard conditions (Table 1). Accurate, reproducible measurements of the k_d_ rate constants were obtained by monitoring the dissociation of WT-FVIII-C2 from BO2C11 (and muteins as appropriate) for 1 hr (S1 Fig.). Substitutions at only six sites resulted in k_d_ values > 4X that of WT-FVIII-C2. The k_a_ and k_d_ rate constants for the binding of WT-FVIII-C2 and for muteins with substitutions distant from the antigen-antibody binding interface were consistent, indicating proper folding of these muteins.

Van’t Hoff analysis allowed quantification of energetic consequences of individual amino acid substitutions. FVIII-C2-R2220A and FVIII-C2-R2220Q could not be evaluated thermodynamically because these substitutions abrogated binding. Although a relative order of energetic contributions was established for the other residues (F2200>F2196 = R2215>N2198>M2199) the *ΔΔG_A_º* values for these substitutions were similar (~11±3 kJ/mol). Four of these five muteins exhibited standard enthalpy values (-15±3 kJ/mol) similar to that of WT-FVIII-C2 (-14 kJ/mol), indicating that the decreased affinity was due to increased entropy of the system (FVIII-C2 mutein + Fab + solvent) (Table 2),e.g. by allowing greater structural flexibility or by changing the solvent exposure of hydrophobic residues and/or the ordering of water molecules. The substitution R2215A had a dramatic effect on the binding enthalpy, reflecting the salt bond between FVIII-C2-R2215 and BO2C11-D52. Interestingly, only one of two beta-hairpin turns in FVIII-C2 that comprise part of the FVIII-Fab interface contributes appreciably to the binding energy (Fig. 1). These results are consistent with an earlier study by Barrow et al. [15]. Variants F2200L and M2199I/F2200L showed dramatically less inhibition by BO2C11, whereas inhibition of the variant L2252F was indistinguishable from that of WT-BDD-FVIII.

The number of well-defined waters in the FVIII-C2 and FVIII-C2-BO2C11 complex crystal structures may indicate entropic contributions of solvent ordering and release. The FVIII-C2 crystal structure includes 46 water molecules within van der Waals distance of the FVIII-C2-BO2C11 interface, and 37 water molecules were built into density at this interface in the FVIII-C2-BO2C11 co-crystal structure, suggesting that release of ~9 water molecules could contribute to the increased entropy that the SPR experiments indicate drives formation of the antigen-antibody complex. Larger amino acid side chains have more degrees of freedom, and their immobilization upon binding to an antibody also contributes to entropic changes.

Substitutions of several amino acids in contact with the antibody (Fig. 1) had little if any effect on the k_d_ or K_D_ values, consistent with the concept of structural versus functional epitopes [43]. Structural epitopes consist of amino acids that are buried in the interface, whereas functional epitopes are the subset of interfacial residues that contribute significantly to the binding affinity. In a seminal paper, Clackson and Wells [44] systematically substituted all interfacial residues on human growth hormone (hGH) and the extracellular domain of its receptor, hGHbp. Interestingly, residues at both molecular surfaces that contributed the most binding energy formed complementary interactions, thus reinforcing the notion of functional epitopes that exist within larger buried surface areas [45, 46]. The importance of determining residence times/avidities, in addition to affinities, is increasingly becoming appreciated in projects to design less antigenic therapeutic proteins [24, 47–49] because prolonged binding of a neutralizing antibody, regardless of its affinity, will block the proper functioning of the antigen. For in-depth analyses, however, the effects of sequence modifications on both avidities and affinities of antibody binding are required.

Mutations at FVIII positions 2196, 2198, 2199, 2200, 2215 and 2220 resulted in diminished binding to BO2C11. The substitution T2197A did not affect the *k*_d_ appreciably, but interestingly, its effect on k_a_ approximately doubled the residence time for this mutein binding to BO2C11 (Table 1). The T2197 hydroxyl moiety forms a weak hydrogen bond with the Y33 hydroxyl group in BO2C11 (d = 3.4 *Å*) [33] and is hydrogen bonded to a water molecule in the FVIII-C2 structure [50]. If Y33 in BO2C11 also forms a hydrogen bond with water in free BO2C11, then one might expect the T2197-Y33 interaction to be entropically favored, as two waters would be released. Thermodynamic data for T2197A indicate that the substitution decreased binding affinity (*ΔΔG_A_º* = +4 kJ/mol) with both entropy (*Δ*(*TΔS_A_º*) = -2 kJ/mol) and enthalpy (*ΔΔH_A_º* = +2 kJ/mol) contributing to the loss. The enthalpic and entropic changes associated with the 5 out of 6 substitutions for which significantly altered *k_d_* values could be measured resulted in energetic costs ranging from 8 to 13 kJ/mol. These estimated contributions to the binding energy (-53 kJ/mol) did not fully account for the standard Gibbs free energy for WT-FVIII-C2 binding to BO2C11 (-68 kJ/mol). Additional binding energy is clearly contributed by R2220 and by additional factors, e.g. changes in flexibility and solvation and smaller contributions from other residues such as T2197.

Substitutions of other residues in intimate contact with the antibody, notably the adjacent β hairpin consisting of S2250-T2253, did not affect measured *k_d_*values appreciably. S2250, which contributed 8 kJ/mol of the total binding energy, forms a hydrogen bond with the antibody D100 side chain carbonyl (d = 2.5 *Å*) and also interacts with the backbone nitrogen of P101 (d = 3.7*Å*). Both FVIII-C2-S2250 and BO2C11-D100 are surface-exposed, so complex formation presumably involves exchange of a hydrogen bond with bulk solvent for an intermolecular bond. It has been suggested that hydrogen bonds contribute about 2.5 kJ/mol to protein folding when folding causes exchange of a protein-solvent hydrogen bond for one that is sequestered within the protein interior [51]. The *ΔΔG_A_º* for FVIII-C2-S2250A was +8 kJ/mol, with an enthalpic change of +11 kJ/mol, which may reflect loss of a hydrogen bond between FVIII-C2-S2250 and BO2C11-D100. Solvent shielding by hydrophobic groups in the interface may strengthen this bond in the WT-FVIII-C2-BO2C11 complex. L2251 and L2252 contact the antibody residues BO2C11-V2, BO2C11-Y27 and BO2C11-L32 when the FVIII-C2-antibody complex is formed [33]. Although the *ΔG_A_º* values for WT-FVIII-C2, FVIII-C2-L2251A and FVIII-C2-L2252A were similar (-68, -66, and-63 kJ/mol, respectively) the corresponding enthalpic and entropic changes were substantial. The binding enthalpy of the mutants with leucines replaced by alanines (*ΔΔH_A_º* = -17 and-35 kJ/mol for L2251A and L2252A, respectively) were much more favorable than for WT-FVIII-C2, possibly due to strengthening of nearby salt bridges and/or hydrogen bonds. However, the substitutions did not change the *K_D_* appreciably because the entropy increase upon binding that drives complex formation for WT-FVIII-C2 was diminished for the muteins (*Δ*(*TΔS_A_º*) = -19 and -40 kJ/mol), compensating for the favorable enthalpic effect of the substitutions. Solvent exposure of L2251 and L2252 would also be expected to cause ordering of nearby water molecules, which would be released upon antibody binding, further driving the binding entropically.

IgG_4_ antibodies such as BO2C11, which have large complementarity determining regions (CDRs) and therefore are likely to shield extensive surfaces of their targets, are common in anti-FVIII immune responses [52, 53]. Many inhibitor antibodies block FVIII binding to activated membranes and VWF, suggesting that they bind to epitopes overlapping that recognized by BO2C11. Our results suggest that a limited number of amino acid substitutions could produce modified FVIII proteins capable of eluding antibodies that bind to similar epitopes. To test this concept directly, BDD-FVIII muteins with the substitutions F2196A, F2196K and M2199A were characterized. These substitutions were chosen because FVIII-C2-F2196A and FVIII-C2-M2199A bound to PS:PC membranes (S2 Fig.), a requirement for FVIII procoagulant activity. BHK clones expressed BDD-FVIII-F2196A at low levels, indicating this substitution might affect protein folding. Lysine occupies this position in the homologous factor V C2 domain, and BDD-FVIII-F2196K was expressed at a level similar to that of WT-BDD-FVIII. BDD-FVIII-M2199A was previously shown to have cofactor activity [54], so it was also chosen for analysis. The F2196K and M2199A variants retained FVIII activity and bound to VWF with affinities similar to WT-BDD-FVIII. Finally, experiments were carried out to test whether rational B-cell epitope modification indeed enabled these BDD-FVIII muteins to avoid neutralization by BO2C11. BDD-FVIII-M2199A had only a ~4X reduced affinity for BO2C11, consistent with BO2C11 inhibition assay results showing only marginal if any protection from inhibition by this mAb (Fig. 4). Similar results were reported earlier by Barrow et al. [15]. The substitution F2196K caused a 28-fold decrease in BO2C11 binding affinity and conferred remarkably complete protection against neutralization by both BO2C11(Fig. 4) and the murine anti-FVIII-C2 mAb 1B5. These results were consistent with an earlier study showing that F2196 contributes significantly to the binding avidity of FVIII-C2 to both of these mAbs [24].

Future studies will evaluate the effects of modifying the amino acid sequence at multiple epitopes comprising functional B-cell epitopes on the FVIII surface [19, 20]. The present study provides proof-of-principle for a strategy to design FVIII variants that can evade immunodominant inhibitors while retaining reasonable procoagulant activity. Although antibodies could eventually be produced against sequence-modified proteins as well, the use of such engineered FVIII proteins could allow clinical stabilization of bleeding while immunosuppression and/or immune tolerance induction are attempted. The present study indicates that SPR is a useful method with which to identify amino acid residues that contribute to epitopes recognized by immunodominant anti-drug antibodies. The combination of rational mutagenesis, measuring the binding affinities and avidities of muteins for anti-drug antibodies, and evaluation of the biological activity of sequence-modified proteins is a promising approach to develop less antigenic FVIII variants with translational potential.

## Supporting Information

S1 Methods Expression and characterization of FVIII-C2 and BDD-FVIII proteins. (PDF)

S1 Table Primers used for mutagenesis of the FVIII-C2 protein. Sequences are all in the 5’-3’ direction. (PDF)

S2 Table Nonlinear fitting of thermodynamic data using the modified Gibbs-Helmholtz equation. (PDF)

S1 Fig A. Sensorgrams of FVIII-C2 wild-type and muteins corresponding to Fig. 1, with the fits to theoretical curves generated using the calculated rate constants overlaid in red. The RU (resonance unit) values are all normalized to zero at the sample injection time point. **B.** Sensorgrams showing the binding of the 6 FVIII-C2 muteins identified as the BO2C11 functional epitope to monoclonal antibody I54, which was shown by competition ELISA experiments to bind to a FVIII-C2 epitope distinct from that recognized by BO2C11. Fits to theoretical curves generated using the calculated rate constants are overlaid in red. The RU values are all normalized to zero at the sample injection time point. These results indicate that the alanine substitutions did not interfere with binding to I54 and hence did not cause significant structural perturbations at FVIII-C2 regions distal from the BO2C11 epitope. **C**. Due to the very slow dissociation of WT-FVIII-C2 from BO2C11-Fab, the kinetic constants were also determined using a differential dissociation time protocol. This protocol was used to decrease the experimental time while providing for the accurate identification of the dissociation rate by using a 1 hr dissociation time. A 3-fold dilution series was used, spanning 20–0.25nM. The replicates for the 2.2nM injection (middle) are superimposed, reflecting the reproducibility of this assay. Fits of the experimental data to a 1:1 model are shown in black. The kinetic constants determined using both standard and differential dissociation time protocols were consistent.
(PDF)

S2 Fig ELISA assays measuring binding of FVIII-C2 proteins to phospholipids. WT-FVIII-C2 and the 2196A and 2199A muteins bound to PS/PC but not to PC in a dose-dependent manner, whereas the substitutions 2198A, 2200A, 2215A and 2220A did not. The apparent binding of FVIII-C2-F2196A to PC was an unanticipated result, possibly indicating that an alanine substitution of this mostly-buried side chain position perturbed the structure sufficiently to cause hydrophobic interactions with the uncharged as well as charged phospholipid surfaces
(PDF)

S3 Fig Characterization of BDD-FVIII muteins. Supernatants from BHK cell cultures grown in serum-free medium were collected and assayed for FVIII expression and activity using a sandwich ELISA (**A**), one-stage clot assay (**B**), one-stage and two-stage clot assays to measure the activation quotient (**C**), and chromogenic assay (**D**). Assays were carried out for untransfected BHK cells and for several lines each of BHK cells expressing WT-BDD-FVIII and the variants BDD-FVIII-F2196A, F2196K, and M2199A. All results are expressed as the mean ± the standard deviation derived from triplicate determinations
(PDF)

S4 Fig Deep purple stained 4–12% NuPAGE Bis-Tris gel showing purity of BDD-FVIII proteins. Gel was run under reducing conditions. Lanes 2–9 contained 100 ng of purified FVIII proteins. In lanes 3, 5, 7, and 9, 100 ng of FVIII protein was digested with 6 U/ml human alpha-thrombin at 37°C for 10 min. Lane 1, Benchmark Protein Ladder; lanes 2 and 3, research grade Kogenate-FS; lanes 4 and 5, WT-BDD-FVIII; lanes 6 and 7, BDD-FVIII-F2196K; lanes 8 and 9, BDD-FVIII-M2199A; lane 10, 6 U/ml human alpha-thrombin
(PDF)
