# Supplementary material for: Six Amino Acid Residues in a 1200 Å2 Interface Mediate Binding of Factor VIII to an IgG4κ Inhibitory Antibody
Source: PLoS One. 2015 Jan 23;10(1):e0116577. doi: 10.1371/journal.pone.0116577 (PMC4304825; doi:10.1371/journal.pone.0116577)
Supplement: S1 Methods — (PDF) [file pone.0116577.s001.pdf]

SUPPLEMENTAL METHODS. Expression and characterization of FVIII-C2 and BDD-FVIII proteins.

Expression of FVIII-C2 proteins

Both wild-type and 43 FVIII-C2 variants having single amino acid substitutions at surface-exposed residues were produced in *E. coli.* The wild-type C2 (WT-C2) sequence, consisting of residues 2170-2332, was amplified from a puc18-C2 plasmid[1] using PCR primers introducing a 5' NdeI restriction site and a 3' BamHI restriction site: forward: 5'-GGCGCGCATATGGATTTAAATAGTTGCAGCATG; reverse: 3'-GGCGCGGGATCCCTAGTAGAGGTCCTGTGC. The PCR product was digested with NdeI and BamHI (New England Biolabs, Ipswich, MA) and subcloned into expression vector pET-16b(+) (Novagen, San Diego, CA), linearized by digestion with the same enzymes, to make pET-16b-WTC2. pET-16b introduces an N-terminal extension of 10 His residues. Mutations to introduce single amino acid substitutions were designed after calculating solvent exposures of all amino acid residues from the FVIII C2 domain crystal structure[2] using the program Stride[3]. Forty-three C2 constructs with a single surface-exposed residue changed to alanine and/or another residue were generated (**Table 1**) using the QuikChange protocol (Stratagene, La Jolla, CA); mutagenesis primers are in **Supplemental Table 1**. The pET-16b-C2 plasmids were purified by minipreps (Qiagen, Valencia, CA) and all C2 sequences verified by DNA sequencing. The host strain *E. coli* OrigamiB(DE3)pLysS (Novagen) was transfected by adding 20 μl of a log phase culture grown in Luria Broth (LB) to 1 μl of each pET-16b-C2 plasmid (miniprep DNA diluted 1:5 in distilled water), incubating this mixture for 30 sec at 42°C followed by 2 min on ice; 80 μl SOC medium was added and cultures were shaken at 37°C for 1 hr and plated on LB/agar plates containing 75 μg/mL carbenicillin, 34 μg/mL chloramphenicol. The plates were incubated at 37°C overnight, then five colonies were picked for each mutant and 10 mL cultures grown overnight in LB plus carbenicillin (75 μg/mL) and chloramphenicol (34 μg/mL). Three mL of each

culture was added to 150 mL LB and shaken at 37°C to log-phase growth, 150 μl 1M IPTG was added, and the culture was shaken for 15-20 min at 37°C, then at 8°C or 16°C overnight. Cells were pelleted at 4000 x g for 20 min, the supernatant discarded, and 4 mL Bug Buster extraction reagent (Novagen) plus 4 μl benzonase (250 U/μl) (Novagen), 0.133 μl rLysozyme (300kU/μl) (Novagen) and 2.5% v/v glycerol was added. Cells were carefully resuspended and stirred for 20 min at room temperature, then centrifuged at 16,000 x g for 30 min at 4°C and the supernatant was applied to a His-Bind purification column (Novagen). The eluate was dialyzed against 4L 20 mM Tris HCl, 150 mM NaCl, 2.5% v/v glycerol, pH 7.4 two times, then against 4L 10 mM HEPES, 150 mM NaCl, 2.5% v/v glycerol, pH 7.4. Sodium azide was added to 0.015% (w/v). Several muteins tended to precipitate, so all samples were spun in a benchtop centrifuge following dialysis for 2 min at 13,000 rpm, and soluble protein in the supernatant was diluted to ~0.1 mg/mL and stored at 4°C. Protein concentrations were determined by Absorbance at 280 nm, using a calculated extinction coefficient of $\varepsilon^{280nm,0.1\%}$ = 1.8[2]. Concentrations of protein solutions with A_{280} < 0.2 were determined using the DC protein microplate assay kit from BioRad (Hercules, CA).

Expression and refolding protocols were optimized for several muteins as follows: (1) after IPTG induction, cell cultures were grown at 4°C overnight and glycerol was not added to the extraction reagent; the His-Bind column eluate was dialyzed against 1L 20 mM Tris HCl, 500 mM NaCl , 0.3% N-lauroylsarcosine, pH 7.9 for 2-3 hr. The buffer was diluted sequentially by adding 500 ml 20 mM Tris HCl, 60 mM NaCl, 0.3% N-lauroylsarcosine, pH 7.9 every 2-3 hr to 4L. The sample was then dialyzed twice against 4L 20 mM Tris HCl, 60 mM NaCl, 0.3% N-lauroylsarcosine, pH 7.9.

Samples were assessed for possible aggregation by size-exclusion chromatography on a Superdex 75 column (GE); each C2 protein eluted as a single major UV peak (>88% of total area under peaks); retention times were compared to those of typical standards run on the

same column (aprotinin: 6.5 kDa, cytochrome c: 12.4 kDa, and carbonic anhydrase: 29 kDa), and each eluted at a position consistent with the C2 monomer size (20 kDa). Larger molecular-weight peaks each comprised < 1% of the total area under peaks in the chromatogram. SDS-PAGE of C2 proteins purified on the nickel column indicated they were ≥ 90% pure[4] so SPR analysis was carried out without further purification. Dynamic light scattering was used to analyze some of the FVIII-C2 proteins as an independent method to confirm they were monomeric in solution.

Binding of FVIII-C2 proteins to phospholipids

Phosphatidylcholine (PC) and phosphatidylserine (PS) were from Avanti Polar Lipids, Inc. (Alabaster, AL). Anti-FVIII-C2 monoclonal antibody ESH8 was from American Diagnostica (Stamford, CT). 96-well Nunc-Immuno polysorp ELISA plates, goat anti-mouse-IgG and rabbit anti-goat-IgG-HRP were from Sigma-Aldrich (St. Louis, MO). Phospholipids were either used immediately after opening the vials or else frozen immediately in aliquots at -80°C that were used within 1 month. Sample buffer for C2 proteins was 10 mM HEPES, 150 mM NaCl, pH 7.4, 0.3% sarcosine, 1.5% glycerol. FVIII-C2 protein stocks were between 0.1 and 5 mg/mL. Buffers for ELISA were as follows: Wash buffer (WB): 50 mM HEPES, 0.15M NaCl, 0.02% Tween-80; Blocking buffer (BB): WB + 0,5% gelatin. PC or a 20:80 molar ratio of PS:PC were diluted to a final phospholipid concentration of 200 μM in methanol and 50 μl were added to wells of 96-well plates. The plates were incubated at room temperature overnight and then blocked with 300μl BB (1 hr 37°C) and washed 3X with 200 μl WB. Fifty μl of WT-FVIII-C2 or FVIII-C2 muteins 2196A, 2198A, 2199A, 2200A, 2215A and 2220A were diluted serially in BB, added to the wells and incubated overnight at room temperature and then washed 3X with WB. ESH8 was diluted 1:2000 in BB, 50μl was added to each well and incubated for 1 hr at 37°C and the wells were washed 3X with 200 μl WB. Anti-mouse IgG-HRP was diluted 1:2000 in BB, 50 μl was added to each well and incubated for 1 hr at 37°C and the wells were washed 3X with 200μl WB. 125μl

TMB substrate (Sigma-Aldrich) was then added to each well and absorbance at 655nm was read in intervals of 1-2 min. Each WT-C2 condition was measured 18X and each FVIII-C2 mutein condition was measured 9X. Results are shown in **Supplemental Figure S2.**

Construction of F8 mutant cDNAs

Amino acid substitutions F2196A, F2196K and M2199A were introduced into BDD-FVIII using splicing by overlap (SOE) extension[5]. The sequence encoding BDD-human FVIII, designated HSQ[6], was contained within the plasmid HSQ/AvrII/ReNeo[7,8]. F8-4162f-P1 (5'-GCTGGGATGAGCACACTTTT-3') and F8-5302r-P4 (5'-CCACCAAAGAAATGCAGGAC-3') primers hybridize at the ends of the target sequence and amplify an ApaI-NotI fragment. The internal primers (designated P2 and P3) contained the mutations. Primers used to produce F2196A were F8-4709f-2196A-P2 (5'-ATTACTGCTTCATCCTACGCTACCAATATGTTTGCCACC-3') and F8-4748r-2196A-P3 (5'-GGTGGCAAACATATTGGTAGCGTAGGATGAAGCAGTAAT-3'). Primers used to produce F2196K were F8-4709f-2196K-P2 (5'-ATTACTGCTTCATCCTACAAAACCAATATGTTTGCCACC-3') and F8-4748r-2196K-P3 (5'-GGTGGCAAACATATTGGTTTTGTAGGATGAAGCAGTAAT-3'). Primers used to produce M2199A were F8-4718f-2199A-P2 (5'-TCATCCTACTTTACCAATGCTTTTGCCACCTGGTCTCCT-3') and F8-4757r-2199A-P3 (5'-AGGAGACCAGGTGGCAAAAGCATTGGTAAAGTAGGATGA-3'). P2 primer was a forward primer facing the NotI site and P3 primer was an exact reverse complement of primer 2. First-round PCR was carried out in two separate reactions with P1/P3 primers and P2/P4 primers. The reaction mix contained 2 ng HSQ/AvrII/ReNeo plasmid DNA, 0.5 μM primers, and 1X Phusion High-Fidelity PCR Master Mix with HP Buffer (New England Biolabs, Ipswich, MA). PCR was performed on a ABI 7900 PCR machine under the following conditions: 1) 98°C, 30 sec; 2) 28 cycles of 98°C, 1 min; 58°C, 1 min; 72°C, 2 min; 3) 72°C, 10 min; and 4) 4°C. PCR products were purified on 1% SeaPlaque agarose gel (Lonza) using a QIAquick Gel Extraction

Kit (Qiagen, Valencia, CA). Second-round PCR was carried out with 25 ng of each first-round PCR product, 0.5 μM P1 and P4 primers, and 1X Phusion High-Fidelity PCR Master Mix with HP buffer using the same PCR conditions as in the first-round PCR. The PCR products and HSQ/AvrII/ReNeo were digested with ApaI (New England Biolabs), column purified (Qiagen), digested with NotI (New England Biolabs), and then purified with the QIAquick Gel Extraction Kit. The digested products were ligated using the Rapid DNA Ligation Kit (Roche, Indianapolis, IN) and transformed into XL-10 gold competent cells (Agilent). Transfection grade DNA was prepared using a Qiagen Plasmid Mini Kit. Constructs were confirmed by sequencing the cloned ApaI-NotI fragments using BigDye Terminator Cycle Sequencing (Applied Biosystems).

BDD-FVIII protein expression

BDD-FVIII and muteins were stably expressed in BHK-M cells as described previously[9]. Briefly, BHK-M cells were cultured with DMEM/F12 medium with L-glutamine and HEPES (Life Technologies, Grand Island, NY) supplemented with 10% fetal bovine serum, 50 units/ml pencillin G, and 50 μg/ml streptomycin sulfate (complete medium). BHK-M cells were transfected with HSQ/AvrII/ReNeo DNA and mutant constructs using Lipofectamine 2000 reagent (Life Technologies). Two days later transfected cells were plated at densities from 1000-5000 cells/10-cm plate in complete medium containing 500 μg/ml Geneticin (G418) (Life Technologies). Colonies were picked when they were large enough to transfer (~2 weeks) to 96-well flat bottom plates containing complete medium with 100 μg/ml G418 (final concentration). When the cells in the well reached confluency, the supernatants were tested for FVIII expression by ELISA. The six lines with the highest FVIII expression for each mutein were kept and expanded further. The FVIII expression levels were then retested and the three cell lines expressing the highest level of each BDD-FVIII protein were selected for further characterization. All cell lines were maintained in complete medium containing 100 μg/ml G418. To measure FVIII activity in cell supernatants, cells were washed with 1X DPBS and transferred

to AIM V® serum-free medium (Life Technologies) when ~70% confluent. Cell supernatant was collected one day later, centrifuged to pellet cells, and supernatants stored as aliquots at -80°C. A number of collections were made until the cells started to detach from the bottom of the flask.

FVIII ELISA

BDD-FVIII expression level in cell supernatants was measured using a sandwich ELISA. Plates were coated with 4 μg/ml anti-human FVIII antibody ESH-4 (American Diagnostica, Stamford, CT) in coating buffer (eBioscience) overnight at 4°C, washed in PBS with 0.05% Tween 20, blocked with diluent solution (eBioscience) for 1 hr at room temperature and washed again. Recombinant FVIII standard (Advate, Baxter Healthcare, Deerfield, IL) or cell supernatant was added to each well and plates were incubated at room temperature for 2 hr and washed. Anti-human FVIII antibody ESH-8 (American Diagnostica) was biotinylated using EZ-Link Sulfo-NHS-LC-Biotin (Thermo Scientific). Biotinylated ESH-8 was added to the plates, incubated at room temperature for 1 hr and washed. Avidin horseradish peroxidase (eBioscience) was added (1:1000 dilution), incubated at room temperature for 30 min and washed. Super Aquablue substrate (eBioscience) was then added and A_{405} measured using a Molecular Devices SpectraMax M5 ELISA plate reader (Sunnyvale, CA). FVIII concentrations were calculated from standard curves (range: 3.9-250 ng/ml) using SoftMax Pro 5.2 software.

FVIII Activity Assays

<u>One-stage assay:</u> FVIII activity was measured using a one-stage coagulation assay. Briefly, 50 μl of standard or sample was added to 50 μl of FVIII-deficient plasma (Siemens), followed by the addition of 50 μl of Dade Actin FSL Activated PTT reagent (Siemens) and incubated for 3 min at 37°C. The reaction was initiated with 50 μl 25 mM $CaCl_2$ and the time required to develop a fibrin clot was measured with a CoaSCREENER. Standard curves were generated using serial dilutions of Dade Behring Control Plasma N (Siemens), which corresponded with 100, 50, 25, 12.5, and 6.25% activity, and analyzed by linear regression analysis of the logarithm of the

clotting time vs. the logarithm of the % FVIII activity. Samples were diluted in 20 mM HEPES, 150 mM NaCl, 0.05% Tween-80, pH 7.4 to a concentration within the standard curve. The calculation of % FVIII activity for each unknown was performed by the CoaSCREENER based on the standard curve entered for each run. Percent FVIII activity was converted to IU/ml by multiplying by the sample dilution and dividing by 100.

Activation quotient: The activation quotient[10] for FVIII expressed in BHK-M cells was measured by determining a ratio for the activity in a two-stage coagulation assay compared with the one-stage coagulation assay. Two cuvettes were set-up, one with 50 μl FVIII sample (cuvette 2) and one without (cuvette 1), and then to both cuvettes was added 50 μl of FVIII-deficient plasma and 50 μl of Dade Actin FSL Activated PTT reagent, which were incubated for 3 min at 37°C. Human alpha-thrombin (Haematologic Technologies, Inc., Essex Junction, VT) was added to a final concentration of 1 U/ml to the FVIII sample diluted in 20 mM HEPES, 150 mM NaCl, 0.05% Tween-80, pH 7.4 and incubated for 40 sec at 37°C. The thrombin activated FVIII sample was further diluted and added to the cuvette without FVIII at 180 sec, followed by initiation of the clotting reaction with 50 μl 25 mM $CaCl_2$. The activation quotient is defined as the ratio of FVIII activity measured by the two-stage assay (cuvette 1) divided by the activity measured by the one-stage assay (cuvette 2). An activation quotient >20 indicates that the FVIII is not significantly pre-activated.

Chromogenic assay: FVIII activity was also measured with the Chromogenix COAMATIC Factor VIII Kit (DiaPharma) and the microplate method was followed according to the manufacturer's instructions.

Purification of BDD-FVIII proteins

BHK-BDD-FVIII expressing cells were expanded in AIM V® serum-free medium in Nunc triple flasks (Rochester, NY) and cell supernatants were collected for purification of FVIII using a two-step ion-exchange procedure[7]. Briefly, Tween-80 (Sigma-Aldrich) was added to the cell

supernatant to 0.01% and loaded onto a SP-Sepharose HP column (GE Healthcare, Piscataway, NJ) equilibrated in 20 mM HEPES, 5 mM $CaCl_2$, 0.01% Tween-80, pH 7.4 (Buffer A) containing 0.15 M NaCl. The column was washed with Buffer A containing 0.22 M NaCl and protein eluted with a 0.22-0.65 M NaCl gradient using an ÄKTAFPLC purification system (GE Healthcare). Fractions containing FVIII were diluted with Buffer A to 0.18 M NaCl. The sample was loaded onto a HiTrap Q HP column (GE Healthcare) equilibrated with Buffer A containing 0.18 M NaCl. The column was washed with Buffer A containing 0.24 M NaCl and eluted with a 0.25-0.8 M NaCl gradient. Fractions were analyzed for FVIII activity using a one-stage coagulation assay. Dilute samples were concentrated with Amicon Ultra-15 centrifugal filter unit with Ultracel-10 membrane (EMD Millipore, Billerica, MA). Concentrations of purified proteins were determined by measuring absorbance at 280 nM and then calculating the concentration using an extinction coefficient of 256,300 $M^{-1}cm^{-1}$.

Gel analysis of purified BDD-FVIII proteins

Purity of samples was analyzed by electrophoresis on 4-12% NuPAGE Bis-Tris mini gel (Life Technologies) before and after digestion with human alpha-thrombin. Purity of BDD-FVIII proteins was compared with research-grade Kogenate-FS, specific activity: 3600 IU/mg (a kind gift from Bayer Healthcare). Samples were digested with 6 U/ml human α-thrombin at 37°C for 10 min. Undigested and digested samples were then mixed with NuPAGE LDS Sample Buffer and Reducing Agent (Life Technologies) to a final concentration of 1X and boiled for 2 min. Samples and molecular weight marker (BenchMark Protein Ladder, Life Technologies) were loaded on the gel and electrophoresed with 1X MOPS SDS running buffer (Life Technologies). The upper chamber buffer contained NuPAGE antioxidant (Life Technologies). Gels were stained with Deep Purple Total Protein Stain according to the manufacturer's instructions (GE Healthcare). Fluorescent staining was imaged with the Typhoon TRIO Variable Mode Imager (GE Healthcare) and viewed with ImageQuant TL (GE Healthcare).

Binding of BDD-FVIII-F2196K and BDD-FVIII-M2199A to VWF

Human VWF (FVIII-free, Haematologic Technologies Inc.) was dissolved in 10 mM acetate pH 5.0 to 10 mg/ml, then immobilized to a CM4 sensor chip using standard amine coupling to a level of 200 resonance units. FVIII and FVIII muteins were injected in a concentration series from 0.22-18 nM (3-fold dilution series) at 50 µl/min for 180 sec using single-cycle kinetics[11]. The association was followed by 600 sec of running buffer (HBS-P^+ and 2 mM $CaCl_2$ pH=7.4) to monitor dissociation. The surface was regenerated with a 40 sec pulse of 3M $MgCl_2$. Duplicate datasets were globally fit to a 1:1 binding model using BiaEvaluation software.

Binding of BDD-FVIII, BDD-FVIII-F2196K and BDD-FVIII-M2199A to BO2C11

The BO2C11 Fab was immobilized on a CM5 biosensor to 20 RU, as described in Methods. Flow cells 1 and 3 were activated, blocked and used as reference cells. BDD-FVIII proteins were injected at 4-5 concentrations resulting in maximum signals of ~5-35 RU and dissociations were monitored for 4,000 sec to determine association and dissociation rate constants at 25°C. Regeneration was accomplished by injecting 10 mM glycine-HCl, pH 2.0 (GE) for 30-60 sec and resonance signals were monitored to ensure complete dissociation of the BDD-FVIII proteins before initiating the next experiment. The sensorgrams were subtracted from the first reference cell signal, subsequently subtracted from the blank run signal and subjected to curve fit analysis using Biacore Evaluation Software with a 1:1 binding model. In order to obtain accurate k_d rate constants for the BDD-FVIII proteins having slow dissociations rates, independent measurements were carried out with longer monitoring of the dissociations, and also using a separate CM5 biosensor with BO2C11 immobilized at 70 RU.

Ability of BO2C11 to inhibit procoagulant activity of BDD-FVIII-F2196K and BDD-FVIII-M2199A

Chromogenic inhibition assay: Briefly, the BDD-FVIII proteins and the mAbs were diluted in human AB serum (Valley Biomedical, Winchester, VA). The diluted BDD-FVIII proteins or pooled normal citrated human plasma (HemosIL calibration human plasma, DiaPharma, West

Chester, OH) were mixed with mAbs at 1:1 ratio (vol:vol), and incubated at 37°C for 2 hrs. As a control, the FVIII samples were mixed with the diluent, human AB serum, for 2 hrs. FVIII activity was measured by a chromogenic functional assay (Coatest SP4 FVIII, DiaPharma), and a standard curve was generated using serially diluted calibration human plasma samples. The extent of inhibition by mAbs was reflected in the residual FVIII activity.

1-stage coagulation inhibition assay: Aliquots of the WT-BDD-FVIII and BDD-FVIII F2196K proteins were thawed and then diluted in human type AB serum to a calculated concentration of 2 U/ml (based on 1-stage activity measurements described above). This concentration, which is in the linear range for the assay, was chosen to better visualize differences in residual activity in the presence of saturating inhibitory antibodies. Equal volumes (300 μl) of BDD-FVIII proteins and 2 μg/ml of neutralizing anti-FVIII mAbs were mixed and incubated at 37°C for 2 hrs. Control samples were pre-incubated with buffer instead of antibody. After the incubation, the FVIII activity in these mixtures was measured using the fully automated STA-R Evolution coagulation analyzer (STAGO) per the manufacturer's instructions. Calculation of the FVIII activity was based on a standard curve generated using serially diluted pooled normal citrated human plasma from Precision Biologic (Dartmouth, NS, Canada) with a concentration of 0.95-1.29 U/ml FVIII. All other reagents, including FVIII-deficient human plasma and PTT reagents, were from STAGO.

SUPPLEMENTAL METHODS REFERENCES

1. Takeshima K, Smith C, Tait J, Fujikawa K (2003) The preparation and phospholipid binding property of the C2 domain of human factor VIII. Thromb Haemost 89: 788-794.
2. Pratt KP, Shen BW, Takeshima K, Davie EW, Fujikawa K, et al. (1999) Structure of the C2 domain of human factor VIII at 1.5 A resolution. Nature 402: 439-442.
3. Frishman D, Argos P (1995) Knowledge-based protein secondary structure assignment. Proteins 23: 566-579.
4. Nguyen PC, Lewis KB, Ettinger RA, Schuman JT, Lin JC, et al. (2014) High-resolution mapping of epitopes on the C2 domain of factor VIII by analysis of point mutants using surface plasmon resonance. Blood 123: 2732-2739.
5. Ho SN, Hunt HD, Horton RM, Pullen JK, Pease LR (1989) Site-directed mutagenesis by overlap extension using the polymerase chain reaction. Gene 77: 51-59.
6. Lind P, Larsson K, Spira J, Sydow-Backman M, Almstedt A, et al. (1995) Novel forms of B-domain-deleted recombinant factor VIII molecules. Construction and biochemical characterization. Eur J Biochem 232: 19-27.
7. Doering CB, Healey JF, Parker ET, Barrow RT, Lollar P (2002) High level expression of recombinant porcine coagulation factor VIII. J Biol Chem 277: 38345-38349.
8. Parker ET, Healey JF, Barrow RT, Craddock HN, Lollar P (2004) Reduction of the inhibitory antibody response to human factor VIII in hemophilia A mice by mutagenesis of the A2 domain B-cell epitope. Blood 104: 704-710.
9. Lubin IM, Healey JF, Scandella D, Runge MS, Lollar P (1994) Elimination of a major inhibitor epitope in factor VIII. J Biol Chem 269: 8639-8641.
10. Lollar P, Fay PJ, Fass DN (1993) Factor VIII and factor VIIIa. Methods Enzymol 222: 128-143.
11. Karlsson R, Katsamba PS, Nordin H, Pol E, Myszka DG (2006) Analyzing a kinetic titration series using affinity biosensors. Anal Biochem 349: 136-147.
