# Supplementary material for: Six Amino Acid Residues in a 1200 Å2 Interface Mediate Binding of Factor VIII to an IgG4κ Inhibitory Antibody
Source: PLoS One. 2015 Jan 23;10(1):e0116577. doi: 10.1371/journal.pone.0116577 (PMC4304825; doi:10.1371/journal.pone.0116577)
Supplement: S1 Table — (PDF) [file pone.0116577.s002.pdf]

**Supplemental Table S1.** Primers used for mutagenesis of the FVIII-C2 protein. Sequences are all in the 5' – 3' direction.

| ***Mutation*** | ***Forward Primer*** |
|---|---|
| S2173I | CTCGAGAAAAGAGTGGATTTAAATGCTTGCAGCATGCCATTGGG |
| E2181A | GCATGCCATTGGGAATGGCGAGTAAAGCAATATCAGATGC |
| F2196A | GCACAGATTACTGCTTCATCCTACGCTACCAATATGTTTGCCACC |
| T2197A | GCTTCATCCTACTTTGCCAATATGTTTGCCACCTGG |
| N2198A | CAGATTACTGCTTCATCCTACTTTACCGCTATGTTTGCCACCTGG |
| M2199A | CTTCATCCTACTTTACCAATGCGTTTGCCACCTGGTCTCCTT |
| F2200A | CTTCATCCTACTTTACCAATATGGCTGCCACCTGGTCTCC |
| A2201P | CCTACTTTACCAATATGTGCGCCACCTGGTCTCCTTCAAAAGC |
| T2202A | CCTACTTTACCAATATGTTTGCCGCCTGGTCTCCTTCAAAAGC |
| K2207A | GGTCTCCTTCAGCAGCTCGACTTCACCTCCAAGGG |
| H2211A | CCTTCAAAAGCTCGACTTGCCCTCCAAGGGAGGAGTAATGCC |
| L2212A | CCTTCAAAAGCTCGACTTCACGCCCAAGGGAGGAGTAATGCC |
| Q2213A | CCTTCAAAAGCTCGACTTCACCTCGCAGGGAGGAGTAATGCC |
| R2215A | CGACTTCACCTCCAAGGGGCGAGTAATGCCTGGAGACC |
| R2220A | CCAAGGGAGGAGTAATGCCTGGGCACCTCAGGT |
| R2220Q | GGGAGGAGTAATGCCTGGCAACCTCAGGTGAATAATCC |
| Q2222A | GGAGTAATGCCTGGAGACCTGCGGTGAATAATCCAAAAGAGTGG |
| V2223M | GCCTGGAGACCTCAGATGAATAATCCAAAAGAGTGG |
| N2224A | GGAGTAATGCCTGGAGACCTCAGGTGGCTAATCCAAAAGAGTGGC |
| N2225A | CCTGGAGACCTCAGGTGAATGCTCCAAAAGAGTGGCTGC |
| K2227A | CCTCAGGTGAATAATCCAGCAGAGTGGCTGCAAGTGG |
| K2227Q | GGAGACCTCAGGTGAATAATCCACAAGAGTGGTGCAAGTGG |
| K2249A | GTAACTACTCAGGGAGTAGCATCTCTGCTTACCAGCATGTATGTG |
| S2250A | CAGGGAGTAAAAGCTCTGCTTACCAGCATGTATGTG |
| L2251A | GAGTAAAATCTGCGCTTACCAGCATGTAT |
| L2252A | CTCAGGAGTAAAATCTCTGGCTACCAGCATGTATGTGAAGG |
| T2253A | GGAGTAAAATCTCTGCTTGCCAGCATGTATGTGAAGGAG |
| H2269A | CATCTCCAGCAGTCAAGATGGCGCTCAGTGGACTCTC |
| Q2270A | GCAGTCAAGATGGCCATGCGTGGACTCTCTTTTTTCAGAATGCC |
| T2272A | GGCCATCAGTGGGCTCTCTTTTTTCAGAATGGC |
| L2273A | GATGGCCATCAGTGGACTGCCTTTTTTCAGAATGGCAAAGTAAAG |
| N2277A | CAGTGGACTCTCTTTTTTCAGGCTGGCAAAGTAAAGGTTTTTCAG |
| K2279A | GGACTCTCTTTTTTCAGAATGGCGCAGTAAAGGTTTTTCAGAATGG |
| P2300L | GGTGAACTCTCTAGACCCACTGTTACTGACTCGC |
| L2302A | GACCCACCGTTAGCGACTCGCTACCTTCGAATTCACC |
| R2304H | CCACCGTTACTGACTCACTACCTTCGAATTCACC |
| R2307Q | CTGACTCGCTACCTTCAAATTCACCCCCAGAGTTGG |
| H2309A | CGCTACCTTCGAATTGCCCCCCAGAGTTGGGTGC |
| Q2311A | CCTTCGAATTCACCCCGCGAGTTGGGTGCACCAG |
| W2313Y | CGAATTCACCCCCAGAGTTACGTGCACCAGATTGCCC |
| H2315A | CCAGAGTTGGGTGGCCCAGATTGCCCTGAGGATGG |
| Q2316A | CCCCAGAGTTGGGTGCACGCCATTGCCCTGAGGATGG |
| E2327A | GGTTCTGGGCTGCGCGGCACAGGACC |

| *Mutation* | *Reverse Primer* |
|---|---|
| S2173I | CCCAATGGCATGCTGCAAGCATTTAAATCCACTCTTTTCTCGAG |
| E2181A | GCATCTGATATTGCTTTACTCGCCATTCCCAATGGCATGC |
| F2196A | GGTGGCAAACATATTGGTAGCGTAGGATGAAGCAGTAATCTGTGC |
| T2197A | CCAGGTGGCAAACATATTGGCAAAGTAGGATGAAGC |
| N2198A | CCAGGTGGCAAACATAGCGGTAAAGTAGGATGAAGCAGTAATCTG |
| M2199A | AAGGAGACCAGGTGGCAAACGCATTGGTAAAGTAGGATGAAG |
| F2200A | GGAGACCAGGTGGCAGCCATATTGGTAAAGTAGGATGAAG |
| A2201P | GCTTTTGAAGGAGACCAGGTGGCGCACATATTGGTAAAGTAGG |
| T2202A | GCTTTTGAAGGAGACCAGGCGGCAAACATATTGGTAAAGTAGG |
| K2207A | CCCTTGGAGGTGAAGTCGAGCTGCTGAAGGAGACC |
| H2211A | GGCATTACTCCTCCCTTGGAGGGCAAGTCGAGCTTTTGAAGG |
| L2212A | GGCATTACTCCTCCCTTGGGCGTGAAGTCGAGCTTTTGAAGG |
| Q2213A | GGCATTACTCCTCCCTGCGAGGTGAAGTCGAGCTTTTGAAGG |
| R2215A | GGTCTCCAGGCATTACTCGCCCCTTGGAGGTGAAGTCG |
| R2220A | ACCTGAGGTGCCCAGGCATTACTCCTCCCTTGG |
| R2220Q | GGATTATTCACCTGAGGTTGCCAGGCATTACTCCTCCC |
| Q2222A | CCACTCTTTTGGATTATTCACCGCAGGTCTCCAGGCATTACTCC |
| V2223M | CCACTCTTTTGGATTATTCATCTGAGGTCTCCAGGC |
| N2224A | GCCACTCTTTTGGATTAGCCACCTGAGGTCTCCAGGCATTACTCC |
| N2225A | GCAGCCACTCTTTTGGAGCATTCACCTGAGGTCTCCAGG |
| K2227A | CCACTTGCAGCCACTCTGCTGGATTATTCACCTGAGG |
| K2227Q | CCACTTGCACCACTCTTGTGGATTATTCACCTGAGGTCTCC |
| K2249A | CACATACATGCTGGTAAGCAGAGATGCTACTCCCTGAGTAGTTAC |
| S2250A | CACATACATGCTGGTAAGCAGAGCTTTTACTCCCTG |
| L2251A | ATACATGCTGGTAAGCGCAGATTTTACTC |
| L2252A | CCTTCACATACATGCTGGTAGCCAGAGATTTTACTCCTGAG |
| T2253A | CTCCTTCACATACATGCTGGCAAGCAGAGATTTTACTCC |
| H2269A | GAGAGTCCACTGAGCGCCATCTTGACTGCTGGAGATG |
| Q2270A | GGCATTCTGAAAAAAGAGAGTCCACGCATGGCCATCTTGACTGC |
| T2272A | GCCATTCTGAAAAAAGAGAGCCCACTGATGGCC |
| L2273A | CTTTACTTTGCCATTCTGAAAAAAGGCAGTCCACTGATGGCCATC |
| N2277A | CTGAAAAACCTTTACTTTGCCAGCCTGAAAAAAGAGAGTCCACTG |
| K2279A | CCATTCTGAAAAACCTTTACTGCGCCATTCTGAAAAAAGAGAGTCC |
| P2300L | GCGAGTCAGTAACAGTGGGTCTAGAGAGTTCACC |
| L2302A | GGTGAATTCGAAGGTAGCGAGTCGCTAACGGTGGGTC |
| R2304H | GGTGAATTCGAAGGTAGTGAGTCAGTAACGGTGG |
| R2307Q | CCAACTCTGGGGGTGAATTTGAAGGTAGCGAGTCAG |
| H2309A | GCACCCAACTCTGGGGGGCAATTCGAAGGTAGCG |
| Q2311A | CTGGTGCACCCAACTCGCGGGGTGAATTCGAAGG |
| W2313Y | GGGCAATCTGGTGCACGTAACTCTGGGGGTGAATTCG |
| H2315A | CCATCCTCAGGGCAATCTGGGCCACCCAACTCTGG |
| Q2316A | CCATCCTCAGGGCAATGGCGTGCACCCAACTCTGGGG |
| E2327A | GGTCCTGTGCCGCGCAGCCCAGAACC |
