# Supplementary material for: Six Amino Acid Residues in a 1200 Å2 Interface Mediate Binding of Factor VIII to an IgG4κ Inhibitory Antibody
Source: PLoS One. 2015 Jan 23;10(1):e0116577. doi: 10.1371/journal.pone.0116577 (PMC4304825; doi:10.1371/journal.pone.0116577)
Supplement: S2 Table — (PDF) [file pone.0116577.s003.pdf]

Supplemental Table S2. Nonlinear fitting of thermodynamic data using the modified Gibbs-Helmholtz equation.

**FVIII-C2 variant**	$\boldsymbol{\Delta H_A^{o\dagger}}$	$\boldsymbol{T\Delta S_A^{o\dagger}}$	$\boldsymbol{\Delta G_A^{o\S}}$	$\boldsymbol{\Delta Cp_A}$	$\boldsymbol{\Delta\Delta H_A^{o}}$	$\boldsymbol{\Delta(T\Delta S_A^{o})}$	$\boldsymbol{\Delta\Delta G_A^{o\S}}$	$\boldsymbol{K_D^{o}}$**(pM)**	$\boldsymbol{K_D}$ **(pM) =** $\boldsymbol{k_d/k_a}$ [‖]
WT-FVIII-C2	-14 ± 4	55 ± 3	-69 ± 1	-0.3 ± 0.9	N.A.	N.A.	N.A.	1	9
**F2196A** [*]	**-29 ± 3**	**27 ± 3**	**-56 ± 1**	**-3.8 ± 1**	**-15 ± 5**	**-28 ± 4**	**13 ± 1**	**154**	**147**
T2197A	-10 ± 4	54 ± 5	-64 ± 1	-1.8 ± 1.3	4 ± 6	-1 ± 6	5 ± 1	6	10
**N2198A**	**-5 ± 8**	**54 ± 9**	**-59 ± 1**	**-4 ± 2**	**9 ± 9**	**-1 ± 9**	**10 ± 1**	**46**	**56**
**M2199A**	**-16 ± 2**	**45 ± 2**	**-60 ± 1**	**-1 ± 0.4**	**-2 ± 4**	**-10 ± 4**	**9 ± 1**	**30**	
**F2200A**	**-22 ± 2**	**33 ± 2**	**-55 ± 1**	**-3 ± 0.5**	**-8 ± 4**	**-22 ± 4**	**14 ± 1**	**230**	**240**
**R2215A**	**-14 ± 5**	**44 ± 5**	**-58 ± 1**	**-2 ± 0.9**	**0 ± 6**	**-11 ± 6**	**11 ± 1**	**68**	**150**
S2250A	-3 ± 5	57 ± 5	-60 ± 1	-3 ± 0.1	11 ± 6	2 ± 6	9 ± 1	30	22
L2251A	-33 ± 15	33 ± 10	-66 ± 1	-0.7 ± 3	-19 ± 16	-22 ± 10	3 ± 1	3	6
L2252A	-49 ± 12	14 ± 12	-63 ± 1		-35 ± 12	-40 ± 12	5 ± 1	9	6

Data are in kJ/mol unless otherwise specified.

[*]Bold-face regions indicate substitutions at the *functional* epitope for BO2C11, i.e. where the substitution caused a greater than fourfold increase in k_d relative to WT-FVIII-C2 binding.

[†]The standard errors (SE) of $\Delta H_{\mathbf{A}}^{\mathbf{o}}$ and $T\Delta S_{\mathbf{A}}^{\mathbf{o}}$ are based on the SE of the slope and intercept, respectively, when the ΔCp_A term is set to zero.

Reported errors for $\Delta\Delta H_A^o$, $\Delta(T\Delta S_A^o)$ and $\Delta\Delta G_A^{o\S}$ are the square roots of the sums of the squares of the errors.

[§]The $\Delta G_{\mathbf{A}}^{\mathbf{o}}$ and $\Delta\Delta G_{\mathbf{A}}^{\mathbf{o}}$ errors were all less than one kJ/mol but are reported here as "±1" for consistency with the significant figures of the measured data.

[‖]The K_D values from the earlier kinetic runs at 25°C only (final column) are included for comparison with K_D values derived from the ΔG_A^o of SPR runs carried out at several temperatures.

Note that the changes in heat capacity, ΔCp_A, were negligible. ΔCp_A values for the L2252A variant are not included because measurements at the lower temperatures produced on and/or off rates that were outside the reliable range for the instrument.
