# Supplementary material for: Six Amino Acid Residues in a 1200 Å2 Interface Mediate Binding of Factor VIII to an IgG4κ Inhibitory Antibody
Source: PLoS One. 2015 Jan 23;10(1):e0116577. doi: 10.1371/journal.pone.0116577 (PMC4304825; doi:10.1371/journal.pone.0116577)
Supplement: S1 Fig — The RU (resonance unit) values are all normalized to zero at the sample injection time point. B. Sensorgrams showing the binding of the 6 FVIII-C2 muteins identified as the BO2C11 functional epitope to monoclonal antibody I54, which was shown by competition ELISA experiments to bind to a FVIII-C2 epitope distinct from that recognized by BO2C11. Fits to theoretical curves generated using the calculated rate constants are overlaid in red. The RU values are all normalized to zero at the sample injection time point. These results indicate that the alanine substitutions did not interfere with binding to I54 and hence did not cause significant structural perturbations at FVIII-C2 regions distal from the BO2C11 epitope. C. Due to the very slow dissociation of WT-FVIII-C2 from BO2C11-Fab, the kinetic constants were also determined using a differential dissociation time protocol. This protocol was used to decrease the experimental time while providing for the accurate identification of the dissociation rate by using a 1 hr dissociation time. A 3-fold dilution series was used, spanning 20–0.25nM. The replicates for the 2.2nM injection (middle) are superimposed, reflecting the reproducibility of this assay. Fits of the experimental data to a 1:1 model are shown in black. The kinetic constants determined using both standard and differential dissociation time protocols were consistent. (PDF) [file pone.0116577.s004.pdf]

Supplemental Figure S1

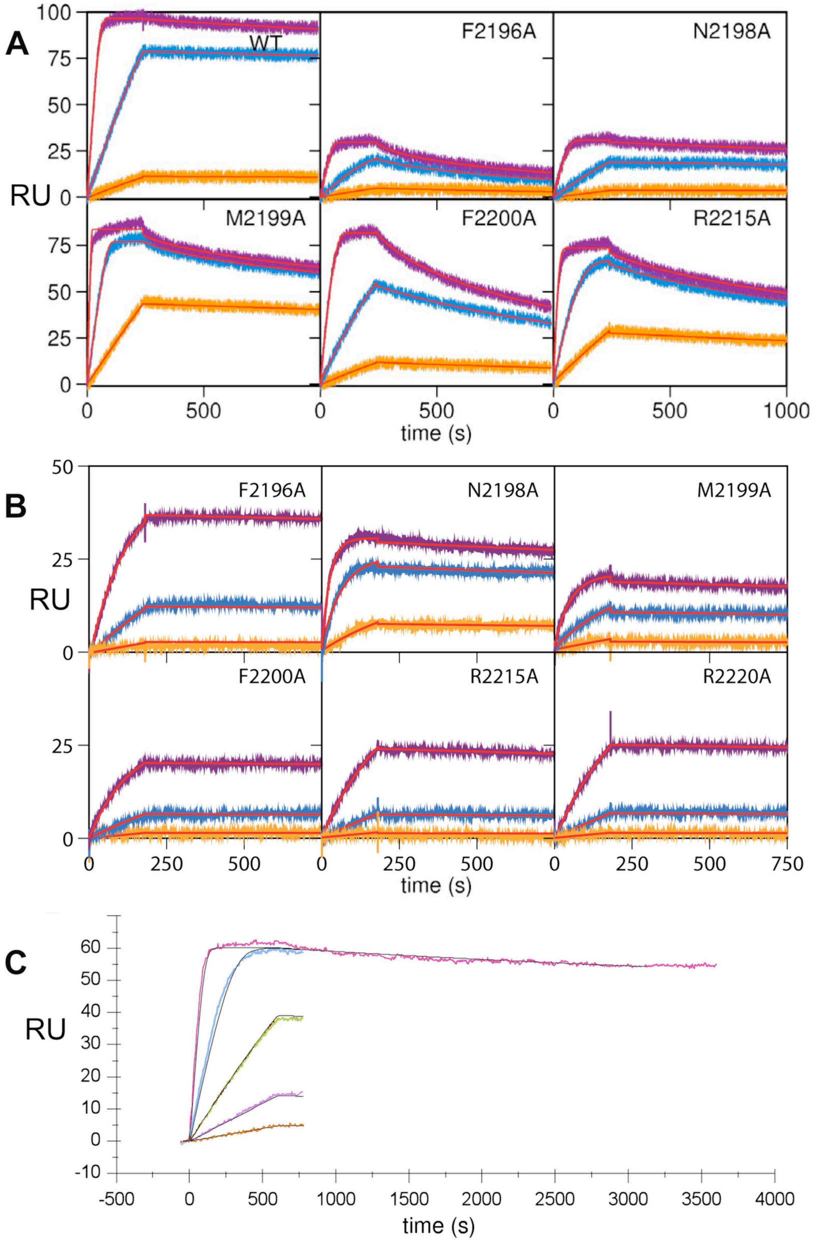

Supplemental Figure S1. A. Sensorgrams of FVIII-C2 wild-type and muteins corresponding to Figure 1, with the fits to theoretical curves generated using the calculated rate constants overlaid in red. The RU (resonance unit) values are all normalized to zero at the sample injection time point. **B.** Sensorgrams showing the binding of the 6 FVIII-C2 muteins identified as the BO2C11 functional epitope to monoclonal antibody I54, which was shown by competition ELISA experiments to bind to a FVIII-C2 epitope distinct from that recognized by BO2C11. Fits to theoretical curves generated using the calculated rate constants are overlaid in red. The RU values are all normalized to zero at the sample injection time point. These results indicate that the alanine substitutions did not interfere with binding to I54 and hence did not cause significant structural perturbations at FVIII-C2 regions distal from the BO2C11 epitope. **C**. Due to the very slow dissociation of WT-FVIII-C2 from BO2C11-Fab, the kinetic constants were also determined using a differential dissociation time protocol. This protocol was used to decrease the experimental time while providing for the accurate identification of the dissociation rate by using a 1 hr dissociation time. A 3-fold dilution series was used, spanning 20-0.25nM. The replicates for the 2.2nM injection (middle) are superimposed, reflecting the reproducibility of this assay. Fits of the experimental data to a 1:1 model are shown in black. The kinetic constants determined using both standard and differential dissociation time protocols were consistent.
