# Supplementary material for: Six Amino Acid Residues in a 1200 Å2 Interface Mediate Binding of Factor VIII to an IgG4κ Inhibitory Antibody
Source: PLoS One. 2015 Jan 23;10(1):e0116577. doi: 10.1371/journal.pone.0116577 (PMC4304825; doi:10.1371/journal.pone.0116577)
Supplement: S2 Fig — WT-FVIII-C2 and the 2196A and 2199A muteins bound to PS/PC but not to PC in a dose-dependent manner, whereas the substitutions 2198A, 2200A, 2215A and 2220A did not. The apparent binding of FVIII-C2-F2196A to PC was an unanticipated result, possibly indicating that an alanine substitution of this mostly-buried side chain position perturbed the structure sufficiently to cause hydrophobic interactions with the uncharged as well as charged phospholipid surfaces (PDF) [file pone.0116577.s005.pdf]

Supplemental Figure S2

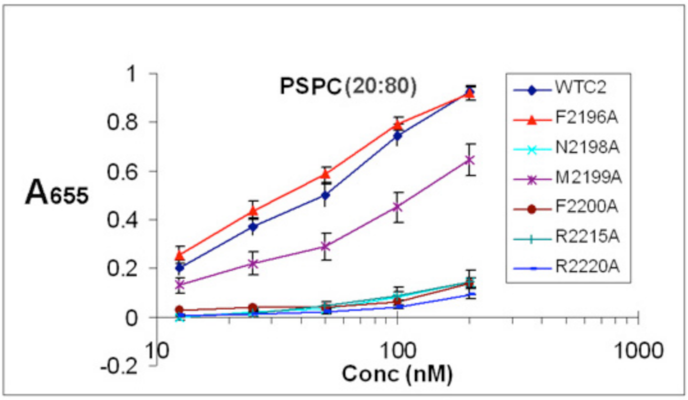

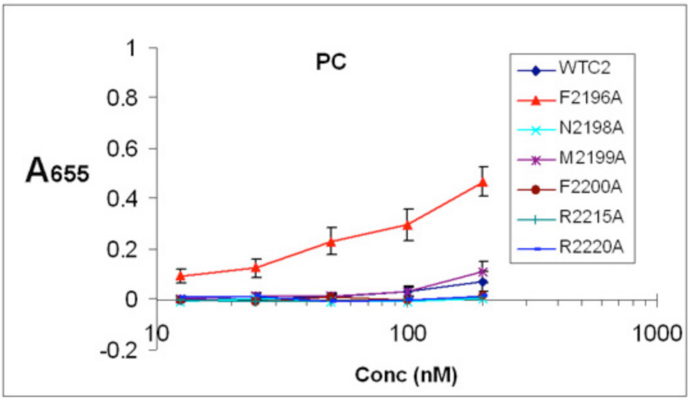

Supplemental Figure S2. ELISA assays measuring binding of FVIII-C2 proteins to phospholipids. WT-FVIII-C2 and the 2196A and 2199A muteins bound to PS/PC but not to PC in a dose-dependent manner, whereas the substitutions 2198A, 2200A, 2215A and 2220A did not. The apparent binding of FVIII-C2-F2196A to PC was an unanticipated result, possibly indicating that an alanine substitution of this mostly-buried side chain position perturbed the structure sufficiently to cause hydrophobic interactions with the uncharged as well as charged phospholipid surfaces.
