# Supplementary material for: Six Amino Acid Residues in a 1200 Å2 Interface Mediate Binding of Factor VIII to an IgG4κ Inhibitory Antibody
Source: PLoS One. 2015 Jan 23;10(1):e0116577. doi: 10.1371/journal.pone.0116577 (PMC4304825; doi:10.1371/journal.pone.0116577)
Supplement: S3 Fig — Supernatants from BHK cell cultures grown in serum-free medium were collected and assayed for FVIII expression and activity using a sandwich ELISA (A), one-stage clot assay (B), one-stage and two-stage clot assays to measure the activation quotient (C), and chromogenic assay (D). Assays were carried out for untransfected BHK cells and for several lines each of BHK cells expressing WT-BDD-FVIII and the variants BDD-FVIII-F2196A, F2196K, and M2199A. All results are expressed as the mean ± the standard deviation derived from triplicate determinations (PDF) [file pone.0116577.s006.pdf]

Supplemental Figure S3

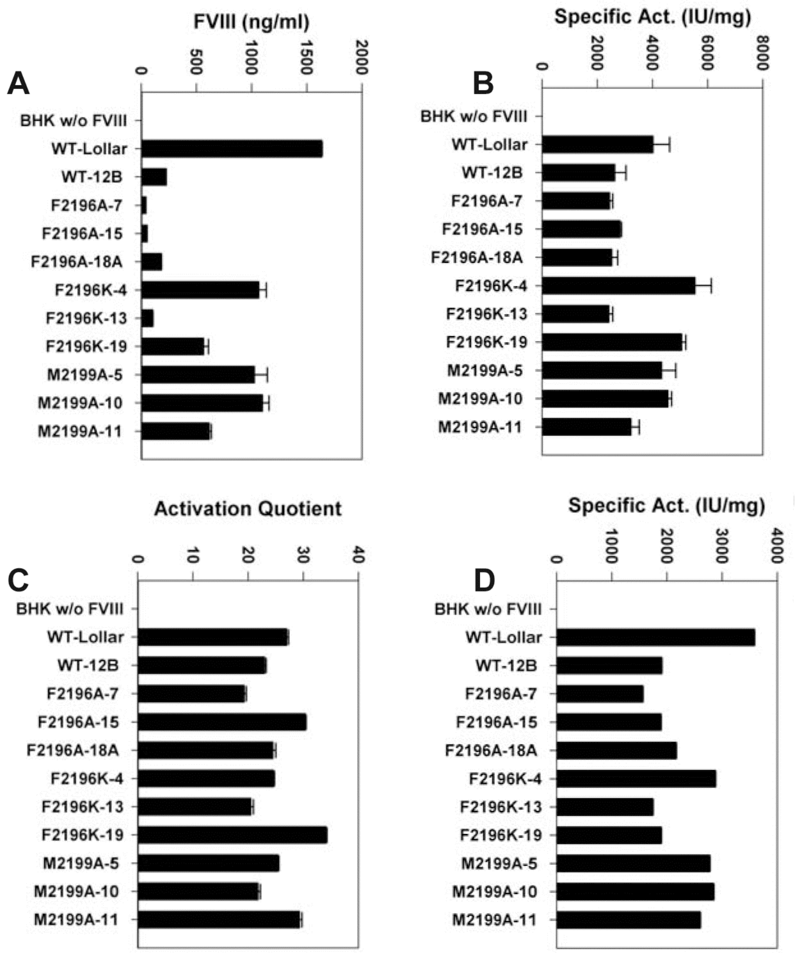

Supplemental Figure S3. Characterization of BDD-FVIII muteins. Supernatants from BHK cell cultures grown in serum-free medium were collected and assayed for FVIII expression and activity using a sandwich ELISA (**A**), one-stage clot assay (**B**), one-stage and two-stage clot assays to measure the activation quotient (**C**), and chromogenic assay (**D**). Assays were carried out for untransfected BHK cells and for several lines each of BHK cells expressing WT-BDD-FVIII and the variants BDD-FVIII-F2196A, F2196K, and M2199A. All results are expressed as the mean ± the standard deviation derived from triplicate determinations.
