# Supplementary material for: Six Amino Acid Residues in a 1200 Å2 Interface Mediate Binding of Factor VIII to an IgG4κ Inhibitory Antibody
Source: PLoS One. 2015 Jan 23;10(1):e0116577. doi: 10.1371/journal.pone.0116577 (PMC4304825; doi:10.1371/journal.pone.0116577)
Supplement: S4 Fig — Gel was run under reducing conditions. Lanes 2–9 contained 100 ng of purified FVIII proteins. In lanes 3, 5, 7, and 9, 100 ng of FVIII protein was digested with 6 U/ml human alpha-thrombin at 37°C for 10 min. Lane 1, Benchmark Protein Ladder; lanes 2 and 3, research grade Kogenate-FS; lanes 4 and 5, WT-BDD-FVIII; lanes 6 and 7, BDD-FVIII-F2196K; lanes 8 and 9, BDD-FVIII-M2199A; lane 10, 6 U/ml human alpha-thrombin (PDF) [file pone.0116577.s007.pdf]

Supplemental Figure S4

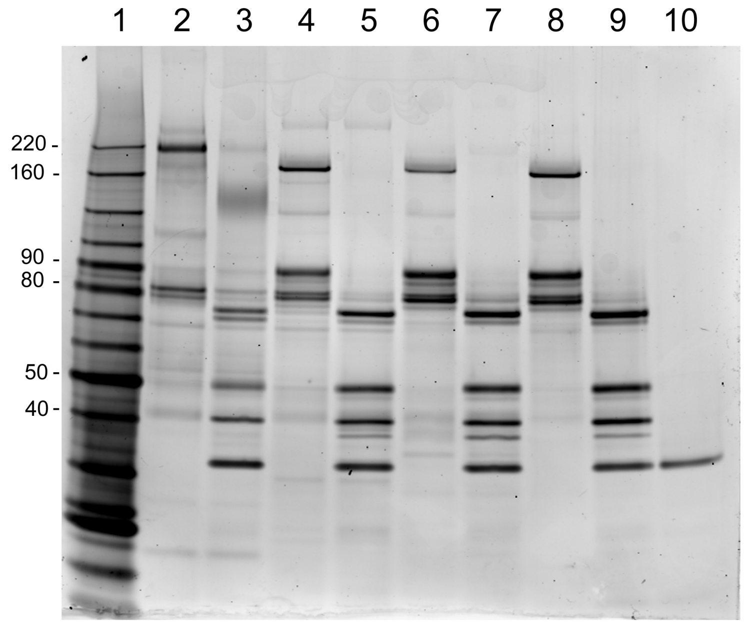

Supplemental Figure S4. Deep purple stained 4-12% NuPAGE Bis-Tris gel showing purity of BDD-FVIII proteins. Gel was run under reducing conditions. Lanes 2-9 contained 100 ng of purified FVIII proteins. In lanes 3, 5, 7, and 9, 100 ng of FVIII protein was digested with 6 U/ml human α-thrombin at 37°C for 10 min. Lane 1, Benchmark Protein Ladder; lanes 2 and 3, research grade Kogenate-FS; lanes 4 and 5, WT-BDD-FVIII; lanes 6 and 7, BDD-FVIII-F2196K; lanes 8 and 9, BDD-FVIII-M2199A; lane 10, 6 U/ml human α-thrombin.
